# The human vagal complex: from gross anatomy to single neurons, from brainstem to abdomen

**DOI:** 10.21203/rs.3.rs-7707775/v1

**Published:** 2025-10-20

**Authors:** Nicole A. Pelot, Ian Baumgart, Ari Blitz, Brandon A.S. Brunsman, Eleana Cintron, Jennifer J. Coleman, Andrew R. Crofton, Jennifer D’Silva, Chris Flask, Aniya Hartzler, Daniel A. Herzka, Proapa Islam, Michael W. Jenkins, Talya A. Jeter, Naomi Joseph, Beverly Koepf, Chaitanya Kolluru, Shruti Kumari, Valerie H. Lam, Leina Lunasco, Michael Markley, Noa B. Nuzov, Ashley Onabiyi, Tatiana Pascol, Rebecca Prince, Zeyna Samba, James Seckler, Anandakumar Shunmugavel, Mallika Singh, Sunah Song, Aniruddha Upadhye, David L. Wilson, Katharine Workman, Jichu Zhang, Andrew J. Shoffstall

**Affiliations:** 1 Department of Biomedical Engineering, Duke University, Durham, NC, USA, 27708; 2 Department of Radiology, Case Western Reserve University and University Hospitals Cleveland Medical Center, Cleveland, OH, USA, 44106; 3 Department of Anatomy, Case Western Reserve University, Cleveland, OH, USA, 44106; 4 Department of Biomedical Engineering, Case Western Reserve University, Cleveland, OH, USA, 44106; 5 Department of Pathology and Cell Biology, University of South Florida, Tampa, FL, USA, 33612; 6 FES Center, Louis Stokes Cleveland VA Medical Center, Cleveland, OH, USA; 7 Department of Pediatrics, Case Western Reserve University, Cleveland, OH, 44106; 8 Cleveland Institute for Computational Biology, Case Western Reserve University, Cleveland, OH, USA, 44106; 9 APT Center, Louis Stokes Cleveland Department of Veterans Affairs Medical Center, Cleveland, OH, USA, 44106

## Abstract

The vagus nerve (VN) connects the brainstem to the viscera to regulate many physiological functions. Mapping the VN with state-of-the-art high-resolution imaging modalities has tremendous potential to improve the safety and efficacy of existing autonomic neuromodulation therapies and to inform design of new therapies. We developed the most comprehensive mapping pipeline of the VN to date, from the perspectives of sample size, lengths of nerve, and range of imaging modalities. Our pipeline maps cadaveric human vagal anatomy with complementary imaging modalities applied serially to the same tissue: pre-dissection MRI, pre-dissection CT, 3D tracing of the dissected nerve *in situ*, CT of the excised nerve, microCT, histology, and a novel serial block-face imaging technique, “3D-MUSE”. The resulting co-registered data enable quantification of gross anatomy, fascicular morphology, and fiber organization in three dimensions. We demonstrated the utility of the data by implementing anatomically realistic computational models of vagus nerve stimulation. These approaches and resulting anatomical data will seed and accelerate the development of novel neuromodulation therapies to treat diseases.

## 1. Introduction

The vagus nerve (VN) is the tenth cranial nerve; it contains sensory and motor nerve fibers that connect the brainstem and viscera, and it serves a critical role across many physiological systems, including cardiovascular, respiratory, digestive, inflammatory, metabolic, and cognitive (Berthoud and Neuhuber 2000; Bonaz et al. 2017; Breit et al. 2018; Johnson and Wilson 2018; Kaniusas et al. 2019). In addition to its inherent physiological roles, the VN is also a key target for neural stimulation therapies: implanted cervical vagus nerve stimulation (VNS) is FDA-approved to treat epilepsy (Handforth et al. 1998), depression (Rush et al. 2005), and stroke sequelae (Dawson et al. 2020); implanted abdominal VNS is FDA-approved to treat obesity (Ikramuddin et al. 2014); and both implanted and non-invasive VNS therapies are under investigation for a wide range of conditions at various points of intervention (Austelle et al. 2024).

Thus, vagal anatomy and its inter-individual variability are foundational for understanding the physiological roles of the VN, for developing neural stimulation devices, and for avoiding nerve damage during surgical interventions, but large and important knowledge gaps remain. For example, with respect to gross anatomy, duplicate cervical VNs have been reported but with unknown prevalence (Labuschagne and Hammer 2019; Márquez-Rivas et al. 2022; Zacharewski and Killory 2023), and cervical VN branching has been examined but did not report the branches’ target organs (Hammer et al. 2015); more broadly, the gross anatomy of the VN is tremendously simplified in textbooks, hampering the ability to avoid nerve damage during surgery and to select rational target locations for neural stimulation therapies. With respect to fascicular morphology—which has important implications for designing neural stimulation therapies (Davis et al. 2023; Musselman et al. 2025)—a recent study revealed that fascicles split and merge in the human cervical VN every 0.56 mm on average (Upadhye et al. 2022); conversely, despite use in preclinical VNS studies, fascicles in pig cervical VNs have a much less plexiform structure (Settell et al. 2020). With respect to ultrastructure, a recent study revealed that some vagal fascicles only contain a few neurons, especially in women, which is the first report of sexual dimorphism in vagal anatomy (Pelot et al. 2020; Havton et al. 2021). Finally, the spatial organization of neurons serving different physiological functions in any given cross section of the human VN is entirely unknown.

We developed a highly innovative pipeline to quantify the vagal anatomy of human cadavers from brainstem to abdomen, spanning gross anatomy to fascicular morphology to ultrastructure, with all branches identified and labeled. We use an array of complementary imaging techniques that are executed serially on the same tissues: computed tomography (CT), magnetic resonance imaging (MRI), *in situ* measurements and photos following dissection, tracing of the three-dimensional (3D) coordinates of the nerve and branches with gross anatomic landmarks, photos and CT of the vagal complex *ex situ*, microCT, histology, and a novel serial block-face imaging method. All data are collected with standardized protocols and detailed documentation, creating the most comprehensive mapping of the VN to date. Designing and executing the pipeline required extensive collaboration of cross-disciplinary teams with expertise in anatomy, physiology, biomedical imaging, and neural engineering, in addition to big-data processing and state-of-the-art machine learning techniques for data analyses.

High-resolution and high-throughput imaging modalities offer a new frontier to generate foundational neuroanatomical datasets, crucial for the development and refinement of autonomic neuromodulation therapies. We are integrating the co-registered segmented images into computational models of VNS to develop therapies with improved efficacy and novel targets, advancing capabilities to selectively activate target fibers while avoiding others that produce unwanted off-target effects. This pipeline and the resulting data and anatomical insights will transform our understanding and treatment of diseases, paving the way for innovative neuromodulation therapies tailored for effective autonomic regulation.

## 2. Results

**Figure 1.**
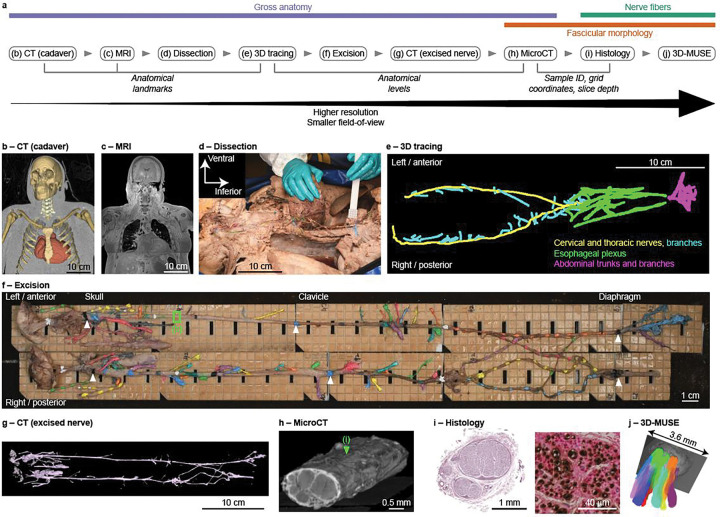
Overview of pipeline for multi-modal imaging of vagus nerves from embalmed human cadavers. Imaging modalities include pre-dissection computed tomography (CT) (b) and magnetic resonance imaging (MRI) (c); digitization of the vagal pathways in situ (e) following dissection of the vagus nerves on the right and left from brainstem to abdomen (d); excision of the complete vagal complex in one piece with the proximal ~1–2 cm of each branch (f), including anatomical “levels” (e.g., white arrowheads on the photo and labels along the top); CT (g) and microCT (h) of the excised nerve; and histology at select locations (i). 3D microscopy with ultraviolet surface excitation (3D-MUSE) (j) uses samples following excision (f). The modalities provide complementary information and their metadata enable automated co-registration (a, italicized labels); image-based refinement of the co-registration between microCT and histology is shown in Figure 3d.

We developed a novel, integrated workflow to comprehensively map the anatomy of cadaveric human VNs from brainstem to abdomen, from gross anatomy to fascicular morphology to ultrastructure (Figure 1). We developed data standards and segmentation methods to handle the large and numerous data files, quantify the anatomy, and parameterize anatomically realistic computational models of VNS. We describe below our approaches for each team and imaging modality, cross-team coordination, and example data outputs.

### 2.1. Anatomical Donation

Cadavers were obtained through Case Western Reserve University’s Anatomical Gift Program (Cleveland, OH, USA). We dissected n=60 cadavers with demographics approximately representative of the US population, including 55% female and 45% male, a distribution of ages (32 to 103 y.o., median of 79.5 y.o.), and a distribution of races and ethnicities, skewed toward White and Black due to Cleveland’s demographics (Supplement 1).

Cadavers were delivered within 2–24 hours after death (except for 3 donors who passed away unobserved at home) and immediately placed in a 35°F cold room. We used a custom formalin-phenol embalming fluid injected within 1 week and typically <72 hours postmortem via the right femoral artery to avoid disturbing the VN; if needed, we delivered additional embalming via the left femoral artery, brachial arteries, and/or carotid arteries, in order of priority. These detailed measures ensured a consistent baseline of tissue integrity, reducing variability and enhancing the quality of downstream imaging and analyses.

### 2.2. CT and MRI

**Figure 2.**
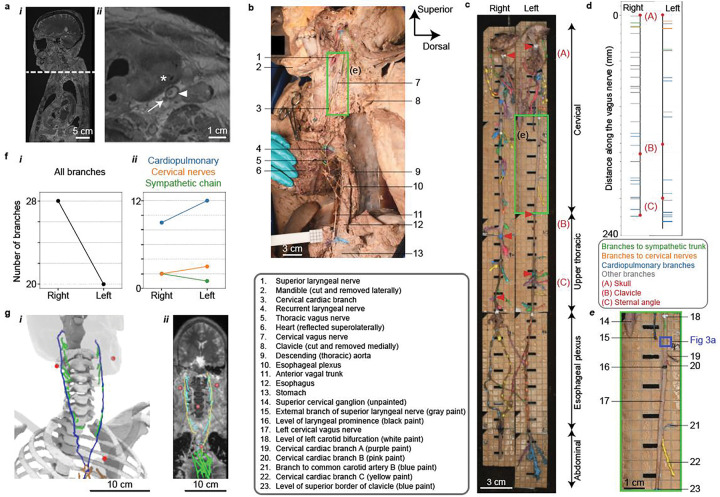
Gross anatomy of the vagus nerve, visualized and quantified via CT, MRI, dissection, and 3D tracing. (a) MRI provides gross anatomical context (i) and some direct visualization of the vagus nerve (ii). The dashed line in panel a-i (sagittal view) denotes the location of the axial cross section in panel a-ii. Arrow: left vagus nerve; arrowhead: left common carotid artery; asterisk: left thyroid lobe. Images are shown for the CISS pulse sequence with 0.5 mm isotropic voxels. (b) Our novel dissection approach enables access to the vagus nerve from brainstem to abdomen with the body supine. The photo shows a lateral view from the left side. (c, e) We glued the excised vagal complex to acrylic boards etched with a 5 mm × 5 mm grid. The green box on panels b, c, and e denotes a matched region of the left cervical vagus nerve. The blue box on panel e denotes the region of the microCT data in Figure 3a. (d, f) We visualized and analyzed the 3D tracing data to quantify locations and counts of vagal branches. The red markers in panels c (arrowheads) and d (dots) denote key axial levels: A, skull = inferior border of the jugular foramen; B, clavicle = superior border of the clavicle; C, sternal angle. Branches to “cervical nerves” in panel f-ii target non-vagal cranial and spinal nerves. (g) Ventral view of the CT (g-i) and MRI (g-ii), providing gross anatomical context to the 3D tracing data, after co-registration based on anatomical landmarks. The red dots denote anatomical landmarks identified in the CT and MRI data (right and left angles of the mandible, jugular notch of the manubrium, and laryngeal prominence (MRI only)).

CT and MRI of the embalmed, undissected cadavers served multiple purposes. The MR images provided a preview of the gross anatomy prior to dissection and provided direct visualization of portions of the cervical and upper thoracic VN (Figure 2a); our advances in MR imaging and analyses provide a foundation for clinical MR-based visualization of the VN. CT scans only required a few minutes, and the skeleton and some soft tissues can readily be segmented from the resulting images. Thus, the CT and MR images both provided a substrate to co-register and visualize the 3D tracing data in gross anatomical context (Sections 2.3 and 2.8, Figure 2g).

Prior to CT and MRI, the cadaver acclimated to room temperature before imaging; otherwise, low body temperature caused poor image quality due to altered radiodensity (Bydder and Kreel 1979; Pandeya et al. 2012), MRI resonance frequency, and MRI relaxation times (Ruder et al. 2012). For CT, the cadaver was placed on the scanner bed and imaged with 300 μm axial slice thickness and in-plane resolution of 0.88 to 0.98 mm (~2–7 min scan time; Siemens SOMATOM Definition Flash Dual-Source CT Scanner). For MRI, after checking for metal using a handheld ferromagnetic target scanner, the cadaver was placed in a plastic vacuum-sealed mattress bag. We secured the cadaver on the scanner table with straps to reduce motion artifacts arising from potential vibrations. We used a 3T clinical scanner (MAGNETOM Vida, Siemens Healthineers, Erlangen, Germany) and multiple receive coils to provide full coverage from the head to the pelvis. We imaged each cadaver with multiple pulse sequences, for 3 to 6 hours of active scan time, achieving sub-millimeter resolutions (0.5 to 0.7 mm isotropic voxels) and complementary tissue contrasts.

### 2.3. Dissection, 3D Tracing, and Removal

We developed a dissection approach to access the complete vagal complex from brainstem to abdomen with all intervening branches—as well as the sympathetic trunk and other nearby cranial and spinal nerves that communicate with the VN—with the cadaver supine throughout the dissection, as detailed in the Methods. Thus, our approach helped preserve anatomical relationships and pioneered the removal of the entire VN complex in one piece.

We exposed the VN through stepwise removal of overlying tissues and organs (Figure 2b, Figure 5). We balanced thorough exposure of the VN with the retention of relevant connective tissue—including small branches that were potentially connective tissue—and adjacent structures to identify nearby nerves that could be targeted or affected by neuromodulation therapies. We photographed the cadaver at each stage and recorded detailed quantitative and qualitative data, including branch metadata (name; orientation along the VN; trajectory in the body; target organs and tissues), caliper measurements of nerve diameters in the coronal and sagittal planes, and dissection notes in a standardized template (Brunsman et al. 2025).

We color-coded each branch and anatomical level (location of a landmark on the VN; e.g., level of the clavicle) *in situ* using tissue dyes (Figure 2b). We developed standardized naming for each nerve, branch, anatomical level, anatomical landmark, and target tissue (Supplement 2), thus ensuring that each label is unique in each subject and that the anatomical terms are fully standardized across subjects and imaging modalities. The VN is highly branched along the lower esophagus and in the abdomen; therefore, we grouped the branches in these regions by general target organ rather than isolating each branch, and in later cadavers, we focused our dissections on the largest branches to enable clearer identification and handling by downstream teams, at the expense of smaller branches.

We collected 3D nerve tracing data following dissection, enabling analysis of branch counts and locations (Figure 2d, f). We used an optical stylus (Polaris Vega ST, Northern Digital, Inc., Waterloo, Ontario, Canada) to digitize the xyz-coordinates of the vagal trunks, each vagal branch, and other dissected nerves connected to the VN. To provide gross anatomic context, we also digitized standardized anatomical “levels” along the VN (e.g., level of the left clavicle on the cervical VN; Supplement 3) and anatomical landmarks in the body (e.g., angle of mandible, sternal angle, aortic hiatus; Supplement 4).

Following 3D tracing, we removed the entire vagal complex in one piece and glued it to a series of acrylic boards (superior-inferior length of ≤9 cm), laser-engraved with a 5 mm × 5 mm grid to define reference coordinates for downstream teams. We annotated a photo of the *ex situ* nerve with all branch names and anatomical levels (Figure 2c, e).

With two dissectors, each cadaver required ~60 to 120 hours of dissection (including photography and note-taking), over 1 to 5 weeks (mean of 2.5 weeks). Color-coding the nerve with tissue dyes, 3D tracing, and removal required an additional ~15 hours per cadaver.

### 2.4. CT of the Excised Nerve

After removal, we stained the VN with 3% phosphotungstic acid (PTA) to achieve clear contrast of the fascicular boundaries while preserving compatibility with immunohistochemistry (Upadhye et al. 2025). Each VN then underwent CT imaging (~1–3 min scan time) (Figure 1g).

### 2.5. MicroCT

**Figure 3.**
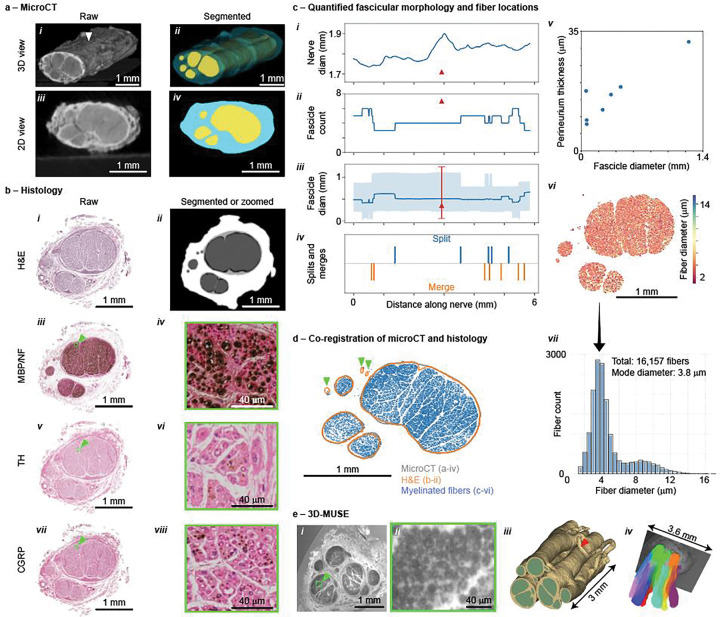
Fascicular morphology and microanatomy of the vagus nerve, visualized and quantified via microCT, histology, and 3D-MUSE. (a) MicroCT shows 3D fascicular morphology. Data shown for the portion of the nerve in the blue box of Figure 2e. The arrowhead marks the approximate location of the histological slices in panel b. Morphology quantified in c-i to c-iv. (b) Histology includes staining with hematoxylin and eosin (H&E; b-i, b-ii; morphology quantified in c-i to c-v) and immunohistochemistry with antibodies against myelin basic protein (MBP/NF; b-iii, b-iv; myelinated fibers quantified in c-vi and c-vii), tyrosine hydroxylase (TH; b-v, b-vi), and calcitonin gene-related peptide (CGRP; b-vii, b-viii). If we define z along the superior-inferior anatomical axis and z=0 for the H&E image, the other cross sections were approximately at z=−4 μm (MBP/NF), 4 μm (TH), and 16 μm (CGRP). (c) We quantified the fascicular morphology using microCT (c-i to c-iv in blue and orange; using data shown in a) and H&E (c-i to c-iii in red; error bars in c-iii mark the range; using data shown in b-i and b-ii). We quantified the perineurium thickness with H&E (c-v). We quantified the diameters and counts of myelinated fibers with MBP (c-vi, c-vii; segmentation and analysis of the image in b-iii). (d) We co-registered the microCT, H&E, and MBP/NF segmentations. The green arrowheads denote fascicles identified by histology but not microCT. (e) 3D microscopy with ultraviolet surface excitation (3D-MUSE) shows 3D fascicular morphology and tractography of fiber groups. The red arrowhead on panel e-iii denotes an example small fascicle in 3D that would not be captured by microCT. Panels b-iv, b-vi, b-viii, and e-ii show zoomed portions of panels b-iii, b-v, b-vii, and e-i, respectively, as denoted by the green boxes and arrowheads. All diameters (nerve, fascicle, fiber) correspond to effective circular diameters, i.e., the diameter of a circle with the same cross-sectional area as contained within the original boundary.

MicroCT enabled visualization and quantification of the complex 3D fascicular structure of the entire *ex situ* vagal complex, as well as its epineurium boundary, providing a critical balance of field-of-view and resolution compared to other imaging modalities (Figure 3a). We cut each VN to separate the “samples” (acrylic boards) and imaged at 11.4 μm isotropic resolution (SCANCO Medical μCT 100; Wangen-Brüttisellen, Switzerland). We imaged two samples (each 9 cm-long) at a time (14 hours). Imaging the whole VN from one cadaver required ~70 to 85 hours of active scan time.

We automatically segmented the fascicles and epineurium using a 3D U-Net (Zhang et al. 2025) (Figure 3a-ii and a-iv). We used the Vascular Modeling Toolkit in 3D Slicer (Izzo et al. 2018) to extract the centerlines of the nerve and branches from the epineurium segmentations. These centerlines served three purposes: (1) defining coordinates to which we attached anatomical labels—including branch names and anatomical levels, (2) defining the coordinates of each branch point along the trunk, and (3) enabling image reslicing along the centerline of each branch and non-vagal nerve to quantify their morphology with transverse cross sections, given that the tissues were oriented obliquely to the imaging planes. From the segmented data, we quantified the nerve diameters, fascicle counts and diameters, and fascicle splitting and merging (Figure 3c-i to c-iv).

### 2.6. Histology

Although histological evaluation of nerve tissue is often considered routine, our project poses unique challenges with respect to tissue quality (embalmed human cadaveric tissue), tissue size (very fine branches), throughput (length of the vagal complex, number of branches, and number of subjects), and the need for images suitable for quantification of anatomical dimensions. We developed a high-throughput, highly documented histological workflow, culminating in the largest and most thorough paraffin-embedded VN tissue repository to date, with ~60 to 220 blocks per cadaver. We meticulously logged each step for robust traceability and facilitating large-scale analyses.

After grossing, embedding, and microtomy, we conducted hematoxylin and eosin (H&E) staining (Figure 3b-i), as well as immunohistochemistry (IHC) with antibodies against myelin basic protein (MBP; Figure 3b-iii, b-iv), neurofilament (NF; to label all axons; Figure 3b-iii, b-iv), tyrosine hydroxylase (TH; to label sympathetic hitchhikers (Kawagishi et al. 2008; Seki et al. 2014); Figure 3b-v, b-vi), and calcitonin gene-related peptide (CGRP; to primarily label afferents; Figure 3b-vii, b-viii). We prioritized certain anatomical locations, including sampling along the cervical and upper thoracic trunks, the recurrent laryngeal nerve, and cardiac branches. We imaged the H&E slides at 20x (0.172 μm pixels) and the IHC slides at 40x (0.087 μm pixels). Automated equipment for processing, embedding, staining, and slide scanning enabled high throughput.

We automatically segmented the inner perineurium, outer perineurium, and epineurium boundaries from each H&E image using a 2D nnU-Net (Isensee et al. 2021) (Figure 3b-ii), and we segmented the fibers identified with IHC using a CellPose model (Stringer et al. 2021) (Figure 3c-vi). From the segmented data, we quantified the fascicular morphology (nerve diameter, fascicle count, fascicle diameters, perineurium thickness; Figure 3c-i, c-ii, c-iii, c-v), as well as the fiber diameters, types, and locations (Figure 3c-vi, c-vii).

### 2.7. 3D-MUSE

We used 3D microscopy with ultraviolet surface excitation (3D-MUSE) (Fereidouni et al. 2017; Kolluru et al. 2022) to achieve detailed 3D visualization of the VN (Figure 3e). 3D-MUSE is a novel modality that acquires a continuous stack of image slices of the whole nerve cross section by iteratively imaging the surface of the tissue block using ultraviolet illumination and removing thin layers. The reconstructed micro-anatomy enables analyses of both 3D morphology (Figure 3e-iii) and fiber pathways (Figure 3e-iv). 3D-MUSE bridges the gap between microCT and standard histology: it provides higher resolution but smaller field of view than microCT, and its resolution is lower than histology, but it provides 3D imaging wherein standard histological images are 2D.

While 3D-MUSE image analysis mirrored microCT capabilities, its distinct contrast and higher resolution enabled three crucial enhancements. First, 3D-MUSE enabled segmentation of both inner and outer perineurium boundaries (yellow in Figure 3e-iii)—rather than only the inner boundary captured by the microCT fascicle segmentations. Second, 3D-MUSE resolved smaller fascicles beyond microCT’s capabilities (red arrowhead in Figure 3e-iii). Third, 3D-MUSE enabled generation of tractograms to map the pathways of fiber groupings through the nerve’s 3D morphology (Figure 3e-iv) (Kolluru et al. 2024).

### 2.8. Co-registration

We co-registered all data for a given subject to leverage complementary anatomical information resulting from imaging the same VN with multiple modalities. We first used metadata for co-registration (Figure 1): standardized anatomical landmarks (CT, MRI, and 3D tracing; Supplement 4), standardized anatomical levels on the VN (3D tracing and microCT; Supplement 3), and grid coordinates of the acrylic backing board and slice depths (microCT, histology). We then further refined the co-registration between microCT and histology using the segmented images and automated algorithms; for the example pair of segmented microCT and H&E images in Figure 3d, the scaling factor of the co-registration algorithm revealed that the microCT fascicles were 8.5% smaller. Thus, the design of our data collection enabled analyses to relate high-resolution, microscopic features (e.g., fascicle arrangement and fiber types) to gross anatomical context for both anatomical investigations and computational modeling of VNS.

### 2.9. Computational Modeling

**Figure 4.**
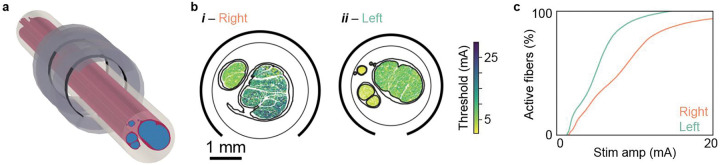
Anatomically realistic, biophysical computational models of vagus nerve stimulation. (a) Finite element model, implemented using the segmented nerve morphology from histology shown in Figure 3b-ii. (b) Heatmaps of activation thresholds for myelinated fibers. Fiber diameters and locations for the model of left vagus nerve stimulation were defined using the MBP segmentation shown in Figure 3c-vi. Both cross sections share the same scale bar and colorbar. (c) Dose-response curves for the thresholds shown in panel b.

Computational modeling and optimization algorithms provide powerful means to design neuromodulation devices that activate targeted nerve fibers mediating therapeutic effects while avoiding activation of off-target nerve fibers mediating side effects. We are using these multi-modal imaging data as inputs to established simulation frameworks (Musselman et al. 2021; Hussain et al. 2024) (Figure 4) and thus implementing a large population of models of human VNS to quantify how inter-individual anatomical differences at the location of stimulation influence neural responses (Figure 4b, c). The CT, MRI, and 3D tracing data enable selection of points of intervention (Figure 2g); the microCT, histology, and 3D-MUSE data define the nerve morphology (Figure 3a-ii, a-iv, b-ii, e-iii; Figure 4a, b); the IHC data define fiber diameters, types, and locations (Figure 3c-vi; Figure 4b), whereas previous models assumed a uniform or synthetic spatial distribution of different fiber diameters in the nerve cross section (Aristovich et al. 2021; Musselman et al. 2023; Romeni et al. 2023); and for modeling true 3D nerve morphology rather than extruded morphology (Marshall et al. 2025b), the 3D-MUSE data define fiber pathways through the fascicular structure (Figure 3e-iv). Implementation and simulation of such numerous and detailed models require automated data querying, automated model parameterization and execution, efficient data handling, and high-performance computing. By integrating these models with optimization algorithms (Wongsarnpigoon and Grill 2010; Aristovich et al. 2021; Hussain et al. 2024), we will design electrode placement, electrode geometry, and stimulation parameters to achieve desired neural responses, ultimately guiding more targeted and effective neuromodulation strategies for the VN.

## 3. Discussion

The VN serves numerous physiological functions and is thus a highly researched target for neuromodulation therapies to treat a wide variety of diseases (Neuhuber and Berthoud 2021; Ottaviani and Macefield 2022; Austelle et al. 2024). However, despite its importance, critical gaps remain in our understanding of vagal anatomy. We developed a rigorous integrated imaging pipeline to quantify the anatomy of the human VN with an unprecedented combination of number of subjects (n=60), anatomical span (from brainstem to abdomen), and range of resolutions (from gross anatomy to fascicular morphology to single neurons). By applying multiple imaging modalities serially to the same tissue, we are generating a foundational resource to understand vagal neuroanatomy and its implications for medical training, understanding and treating dysautonomia, avoiding neural damage during surgery, identifying novel points of intervention for vagal neuromodulation with associated surgical approaches, and model-based design of VNS therapies.

Our quantification of human vagal anatomy builds upon previous studies that had more limited scopes, with fewer subjects, few—often single—anatomical regions, and isolated imaging techniques. For example, numerous cadaver dissection studies investigated vagal branching, such as pathways of cardiac branches of the VN and their connections to cardiac branches of the sympathetic trunk (Mizeres 1963), which are important in VNS to treat heart failure (Ardell et al. 2015; Anand et al. 2020), and the number of abdominal trunks (Doubilet et al. 1948), which impacts implantation of VNS devices to treat obesity (Ikramuddin et al. 2014). Fascicular structure is a key factor affecting VNS activation thresholds (Davis et al. 2023; Musselman et al. 2025); recent histological and microCT studies revealed tremendous variability in morphology of the VN both between individuals and along a given VN (Pelot et al. 2020; Upadhye et al. 2022). Numerous strategies exist for selective nerve stimulation (Fitchett et al. 2021) to improve efficacy and reduce side effects of neuromodulation therapies, and they rely on knowledge of the locations and diameters of target fibers; electron microscopy studies generated histograms of fiber diameters (Schnitzlein et al. 1958; Havton et al. 2021), but the fibers’ spatial organization with associated target organs and physiological functions are unknown. The data from our high throughput, integrated pipeline build upon prior studies of vagal anatomy: (1) we are examining a large population of VNs to provide a more complete view of inter-individual variability and sex differences, (2) we are dissecting and imaging the VN throughout the neck and torso to fill gaps in prior anatomical data and to examine anatomical differences across locations in a given individual, and (3) we are co-registering our imaging data across modalities to examine correlations of multi-scale anatomical metrics.

Following segmentation, we are using the imaging data to parameterize computational models of VNS with unprecedented anatomical accuracy, detail, and inter-individual variability. Multiple open-source software packages exist to standardize and automate anatomically realistic modeling of peripheral nerve stimulation (Lubba et al. 2019; Eiber et al. 2021; Musselman et al. 2021; Couppey et al. 2024). Such computational models synthesize our best understanding of the target system, enable examination of mechanisms of action, and enable global optimization to design VNS therapies over parameter ranges that are infeasible to evaluate in animal or clinical studies. However, the power of computational models relies on appropriate anatomical inputs. Models of peripheral nerve stimulation typically use small sample sizes—often n=1—with simplified or synthetic morphologies with a constant cross section (e.g., (Helmers et al. 2012; Bucksot et al. 2019; Musselman et al. 2023)), although the fascicles of some peripheral nerves—including the human VN—split and merge over short distances (Jabaley et al. 1980; Upadhye et al. 2022). Further, such models also typically assume a uniform distribution of fiber types and diameters over the cross section, although studies of pig VNs show non-uniform distributions (Settell et al. 2020; Kronsteiner et al. 2022; Jayaprakash et al. 2023; Thompson et al. 2025); spatial organization of nerve fibers has important impacts on device design for selective activation (e.g., (Fisher et al. 2013; Dali et al. 2019; Aristovich et al. 2021; Romeni et al. 2023; Hussain et al. 2024)). The large population of models of human VNS built upon these imaging data will provide substantial advances in model-based design of more effective neural stimulation therapies with decreased side effects.

The high throughput and rigor of our data collection relied upon (1) tremendous cross-team collaboration, (2) development and consistent use of standards, and (3) state-of-the-art data handling. We developed detailed protocols for tissue handling, staining, and imaging (see Methods), and we used a communal spreadsheet for recording detailed metadata. We annotated the data with standardized anatomical terms (Supplement 2). We developed data standards for naming and organizing the files consistent with the SPARC Data Structure (SDS) (Bandrowski et al. 2021). Table 1 summarizes our file types and sizes; we used both established (e.g., DICOM) and state-of-the-art (e.g., Zarr^1^, OME-TIFF^2^) file formats, as appropriate for each imaging modality. We set up large data servers with automated backups, we used Globus^3^ (University of Chicago, USA) and gigabit ethernet connections for data transfer, and we used the Pennsieve API^4^ to upload the data for sharing on sparc.science^5^. We set up a GitLab^6^ group for code versioning, internal sharing, and backup, consistent with recommended practices in scientific computing (Wilson et al. 2014, 2017; Taschuk and Wilson 2017; Pelot et al. 2025b). We used state-of-the-art machine learning techniques for automated segmentation (Ronneberger et al. 2015; Isensee et al. 2021; Stringer et al. 2021; Ravi et al. 2024; Zhang et al. 2025). Thus, our highly standardized practices for data collection, organization, curation, and annotation were essential for fully automated data analyses, code-based data querying (e.g., “all microCT and histology images of the left cervical trunk, 5 cm superior to the clavicle, ±5 mm”), and synthesis across subjects.

Primary challenges in mapping human vagal anatomy in a large population included variable tissue quality, trade-offs between imaging modalities, uncertainty in anatomical dimensions and relational anatomy, and anatomical complexity. Tissue preservation, staining, and resulting image quality were variable and unpredictable across subjects, locations, and modalities. Tissue quality could be affected by various factors, including time from death to embalming, embalming method, and air exposure. Subpar tissue quality could impact dissection (especially for small branches), histological data collection (e.g., difficulty in grossing and embedding the smallest branches, tissue slices falling apart, faint antibody labeling, myelin unwinding), and accuracy of automated segmentation pipelines across images with variable staining (given that manual verification of all segmentations was infeasible for such large data quantities). We worked to balance the data collection protocol that achieved the best image quality for a given modality with compatibility with downstream approaches; for example, the staining required for microCT and 3D-MUSE can compromise subsequent histological assays (Upadhye et al. 2025c). Other confounds affected gross anatomy (e.g., 3D tracing after the anatomy was displaced during dissection), as well as nerve size, fascicular morphology, and micro-anatomy; embalming and other tissue processing (dehydration and heating for paraffin embedding) can cause shrinkage and distortion (Hursh 1939; Stickland 1975; Schulz et al. 2011). Finally, human vagal anatomy is highly complex and variable across individuals, which posed numerous challenges across all teams, especially to maintain standardization and high throughput.

Additional challenges included subject demographics, defining scope, and missing data. First, our donor pool is demographically broad but skewed toward older White and Black individuals (Supplement 1), limiting the generalizability of our findings to younger populations and to other races and ethnicities. However, gross anatomy and fascicular morphology are not expected to change with age, and many VNS therapies under investigation target age-related conditions, such as rheumatoid arthritis (Peterson et al. 2024), heart failure (Anand et al. 2020), dementia (Kamoga et al. 2024), and Parkinson’s disease (Farrand et al. 2020). Second, the project’s timeline required some restrictions on data collection scope, particularly for dissection and histology. We prioritized proximal dissection of each branch with sufficient distal dissection to identify branch targets. We selected four primary antibodies for IHC analyses, but antibodies may not exclusively or reliably bind to their canonically targeted fiber types, antibodies are typically developed and characterized for brain tissues, not peripheral nerves, and antibodies are most often used to label cell bodies, not axons. Finally, although our datasets are mostly complete, missing data occurred due to poor embalming, human errors, and equipment maintenance.

Our pipeline enables comprehensive neuro-anatomical characterization that can be leveraged to study any peripheral nerve, with an unprecedented range of anatomical scales. Correlating these detailed anatomical insights with clinical outcomes—such as VNS efficacy for a given target disease—will be key. By integrating high-resolution imaging data into prospective trials and iterative device design, researchers can more accurately target the nervous system. Our approaches lay the groundwork for the next generation of neuromodulation therapies: anatomically informed, computationally optimized interventions that selectively target specific vagal fibers while minimizing unintended off-target effects for robust clinical outcomes. The integration of novel imaging modalities, rigorous data management, model-based design, and data sharing positions this pipeline as a transformative resource for understanding and modulating the autonomic nervous system.

## 4. Online Methods

Our methods for tissue handling and imaging of cadaveric human vagus nerves across multiple imaging modalities are described below and published on protocols.io (Table 2). We use the following terms with specific definitions:

1. Anatomical landmark: Standardized locations in the body to contextualize the 3D tracing data and to enable co-registration to the CT and MRI (e.g., angle of mandible).
2. Anatomical level: Axial level of an anatomical structure on the trunk of the vagus nerve (e.g., level of the left clavicle on the cervical VN).
3. Sample: The VN on each ≤9 cm-long gridded acrylic board (Figure 2c, e).

### 4.1. Anatomical Donation and Embalming

Cadavers (n=60, n=33 F / n=27 M, 32 to 103 y.o.; Supplement 1) were obtained through Case Western Reserve University’s Anatomical Gift Program, excluding donors with trauma to regions containing the vagus nerve (VN), excessive post-mortem time before embalming (>96 hours), and major infectious diseases (e.g., COVID, HIV/AIDS, tuberculosis). This study was determined to be exempt from human subjects research by Case Western Reserve University’s Institutional Review Board (IRB) because it involved de-identified cadaveric tissue and no protected health information was collected. Race and ethnicity were self-reported in an open-text field by donors during the registration process for body donation. Cause of death was obtained from the physician signatory on each cadaver’s death certificate or other healthcare provider if the donor passed in a healthcare institution.

Cadavers were delivered 2–24 hours after death—except for 3 donors who passed away unobserved at home, in which case delivery was within 24 hours of discovery of the body—and immediately placed in a 35°F cold room. We used a custom formalin-phenol embalming fluid injected within 1 week and typically <72 hours postmortem via the right femoral artery to avoid disturbing the VN; if needed—as indicated by excessive joint mobility, poor tissue graying, or supple tissue—we delivered additional embalming via the left femoral artery, brachial arteries, and/or carotid arteries, in order of priority (Nuzov et al. 2025a).

All example data are shown for SR042 (F, Black, 36 y.o.), except the MRI data in Figure 2a (SR044, F, White, 81 y.o.) and the 3D-MUSE data in Figure 1j and Figure 3e (SR001, F, White, 82 y.o.).

### 4.2. CT and MRI

We checked for metal using a handheld ferromagnetic target scanner (FerrAlert^™^, Kopp Development, Inc., Jensen Beach, FL, USA) to ensure MR compatibility, and we placed the cadaver in a vacuum-sealed plastic mattress bag to remove extraneous air, to reduce odor, fumes, and chemical solution exposure, and to maintain a clean imaging environment (Nuzov et al. 2025a).

Prior to CT and MRI, each cadaver was acclimated to room temperature. Each cadaver underwent CT imaging (SOMATOM Definition Flash, SIEMENS, Munich, Germany) with 300 μm axial slice thickness and in-plane resolution of 0.88 to 0.98 mm (Nuzov et al. 2025d). For MRI, each cadaver was secured on the scanner table with straps to reduce motion artifacts arising from potential vibrations. We used a 3T scanner (MAGNETOM Vida, Siemens Healthineers, Erlangen, Germany) and multiple receive coils (Siemens Healthineers, Munich, Germany) to provide full coverage from head to pelvis (Herzka et al. 2025): a standard 20-channel head and neck coil, two 18-channel body coil arrays placed with slight overlap over the trunk, and a 36-channel spine coil. We imaged each cadaver with multiple pulse sequences (Table 3). We manually defaced the CT and MRI data.

### 4.3. Dissection

**Figure 5.**
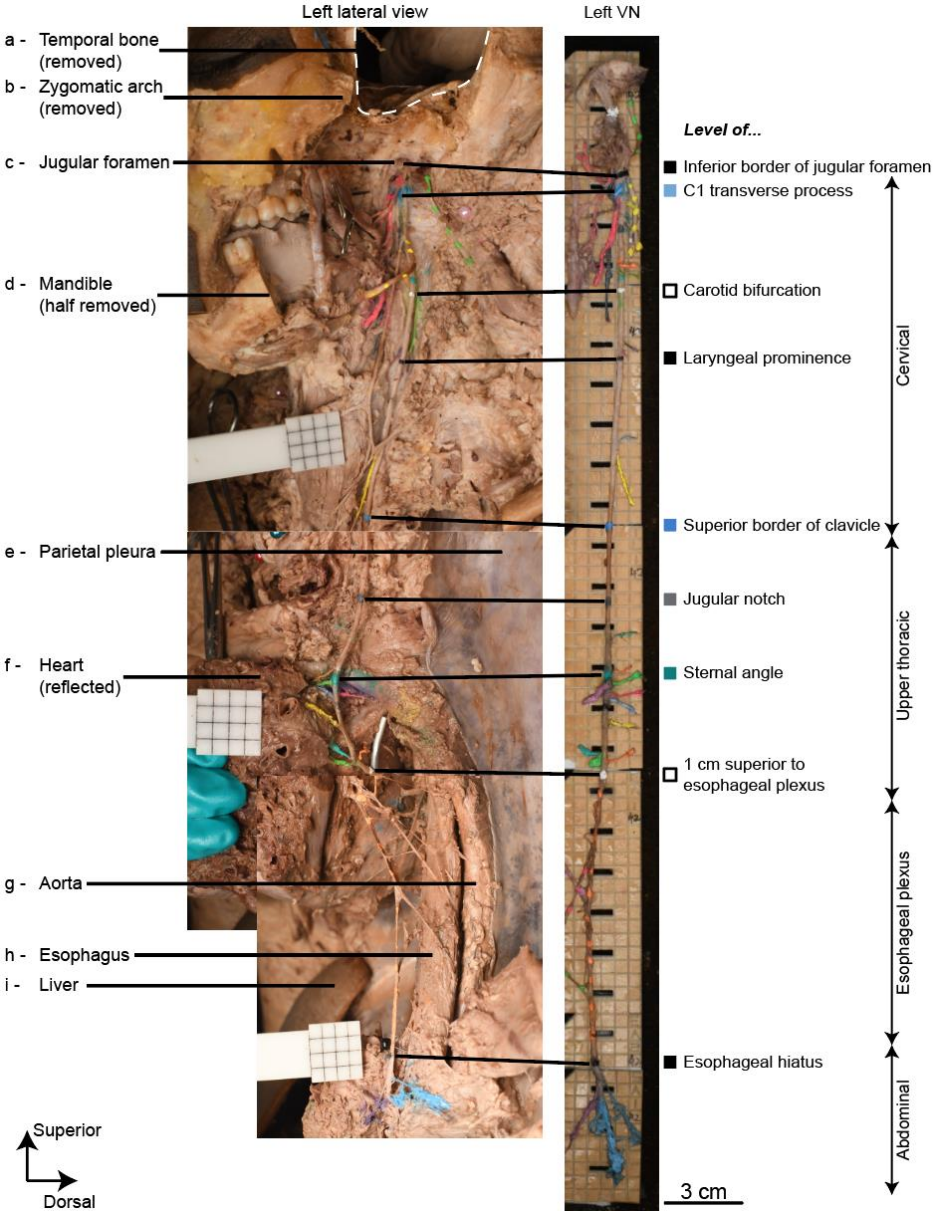
Dissection of the human vagal complex, from brainstem to abdomen, with the cadaver supine. The grids are all 5 mm × 5 mm.

We developed a dissection approach to access the complete vagal complex from brainstem to abdomen, including all intervening branches—as well as the sympathetic trunk and other nearby cranial and spinal nerves that communicate with the VN—with the cadaver supine throughout the dissection (Figure 5). We focused our dissections on the proximal ~2 cm of each branch with sufficient distal dissection to identify each branch’s primary targets.

Our novel dissection approach is detailed in (Brunsman et al. 2025). Briefly, in the cervical region, after removing the skin and superficial structures, we removed the ramus and angle of the mandible (Figure 5d), zygomatic arch (Figure 5b), calvarium, forebrain, and cerebellum, while leaving the brainstem intact below the rostral midbrain. We opened the jugular foramen (Figure 5c) via an anterolateral approach to access the brainstem and rootlets with the cadaver supine, thereby reducing cadaver repositioning that could disrupt relative locations of anatomical structures; specifically, we created a U-shaped opening in the temporal bone slightly anterior and posterior to the external auditory meatus, extending from the skull base to the axial cut made when removing the calvarium (Figure 5a). We dissected the cranial and cervical VN, its branches, and other nearby nerves that have communicating branches with the VN.

In the thorax, after removing the skin and anterior chest wall, including some of the parietal pleura (Figure 5e), we removed the lungs at the roots, lateral to the VN. We removed the heart from the pericardial sac but left the heart *in situ* to preserve cardiac branches (Figure 5f). We dissected the thoracic VN, its branches, and the esophageal plexus.

In the abdomen, after removing the skin, we incised and reflected the parietal peritoneum and mobilized the gastrointestinal viscera. We dissected the distal esophageal plexus, the abdominal trunks, and their branches.

To reduce tissue drying during dissection and thus to preserve tissue quality for downstream imaging, we sprayed the cadaver with a wetting solution of 0.01% liquid phenol (≥89% phenol) and 40% professionalgrade concentrated bleach (8.25% sodium hypochlorite) (v/v) in water, every ~15 minutes while dissecting or more often if tissues appeared to be desiccating. When not in active dissection, we sprayed the cadaver with the same wetting solution, covered the exposed tissue with wetted cheesecloth, and sealed the cadaver in a body bag.

We used premium white latex paint (Rust-Oleum, Vernon Hills, IL, USA) mixed with Davidson Marking System dyes (Bradley Products, Inc., Bloomington, MN, USA) for *in situ* color-coding of each branch and each anatomical level on the VN. We developed standardized naming for each nerve, branch, anatomical landmark, anatomical level, and target tissue (Supplement 2), thus ensuring that each anatomic label is unique in each subject and that the anatomical terms are fully standardized across subjects and imaging modalities.

Our dissection process generated five primary data outputs per cadaver, as detailed in (Brunsman et al. 2025): (1) dissection notes in a standardized template; (2) 52 standardized photographs before, during, and after the dissection; (3) recorded direction of each branch (orientation around the VN and trajectory in the body when leaving the VN); (4) the name and target tissues of each branch (with associated paint color); and (5) at standardized levels, the diameter of the VN and distances to the corresponding anatomical landmarks in the coronal and sagittal planes. We measured nerve diameters with Castroviejo calipers (Sklar, West Chester, PA, USA) for smaller nerves or 6-inch digital calipers (VWR, Radnor, PA, USA) for larger nerves.

### 4.4. 3D Tracing

Our tracing protocol is detailed in (Nuzov et al. 2025b). After dissecting the VN and before removing it from the cadaver, we used an optical stylus (Polaris Vega ST, Northern Digital, Inc., Waterloo, Canada) to digitize the xyz-coordinates of the vagal trunks, vagal branches, and other dissected neural pathways that are connected to the VN. To provide gross anatomic context and enable co-registration to the MRI data, we also digitized standardized anatomical “landmarks” in the body (e.g., the angle of mandible, sternal angle, aortic hiatus; Supplement 4) and anatomical “levels” along the VN (e.g., the level of the left clavicle on the cervical VN; Supplement 3). We acquired the tracing data with NDI ToolBox software (v5.004.015 or v6.001.027 for Windows (64-bit)). For the nerves and branches, we post-processed the data to trim high-density point clouds and fit a B-spline curve to each trace. We defined the point coordinate for each level and landmark as the centroid of the collected point cloud.

### 4.5. Removal of the VN from the Cadaver

Our novel dissection approach enabled removal of the entire vagal complex—from brainstem to abdomen on both the right and left sides—in one piece, as detailed in (Nuzov et al. 2025c). We glued the VN to a series of acrylic boards, laser-engraved with a 5 mm × 5 mm grid to define reference coordinates for histology and 3D-MUSE (Figure 1f; Figure 2c, e); each board was 3 cm wide (medial-lateral) and up to 9 cm long (superior-inferior), where the length was constrained by the bore size of the microCT scanner. We also created holes along the center of the board to improve the uniformity of staining with phosphotungstic acid (PTA).

The VN on each ≤9 cm-long acrylic board was termed a “sample”. As detailed in (Pelot et al. 2025a), we used an identifier (e.g., SR008-CL1) in a standardized format to label each sample uniquely across all samples and cadavers:

1. “SR”, which stands for “SPARC REVA”, i.e., the NIH program that funded this project
  - SPARC: Stimulating Peripheral Activity to Relieve Conditions
  - REVA: REconstructing Vagal Anatomy
2. Three-digit cadaver identifier (001 – 060)
3. Dash
4. Two uppercase letters designating the anatomical region
  - CR: cervical right
  - CL: cervical left
  - TR: thoracic right
  - TL: thoracic left
  - EA: esophageal plexus, anterior
  - EP: esophageal plexus, posterior
  - SA: inferior to the esophageal plexus (sub-esophageal), anterior
  - SP: inferior to the esophageal plexus (sub-esophageal), posterior
5. One digit to enumerate the samples in a given anatomical region

Note that we used “sub-esophageal” in lieu of “abdominal” because the inferior end of the esophageal plexus can be in the thorax or in the abdomen, depending on the cadaver.

We used superglue (Micro Precise Super Glue, Gorilla Glue, Cincinnati, OH, USA) to secure the VN to the acrylic boards at the superior and inferior ends of each board and at the distal end of each branch, with additional glue as needed to secure the esophageal plexus and abdominal samples. We annotated a photo of the *ex situ* nerve with all branch names and anatomical levels (Figure 2c, e).

To avoid movement during transportation, we used waterproof Velcro to secure each acrylic board to a large acrylic sheet, and we used additional Velcro to secure the large acrylic sheet in a plastic container. To avoid dehydration, we covered the nerve with cheesecloth soaked in phosphate-buffered saline and ensured that the container was airtight.

### 4.6. PTA Staining

PTA staining provided contrast between the fascicles and surrounding epineurium for computed tomography (CT) and microCT imaging, as detailed in (Upadhye et al. 2025b, 2025c). We immersed the nerve in 10% formalin for ~12 hours to 4 days between the completion of dissection and the start of PTA staining. For PTA staining, we selected incubation times based on tissue thickness to achieve clear contrast at the fascicle boundaries while preserving tissue integrity; specifically, we stained cervical and upper thoracic samples for 24 hours, and we stained esophageal plexus and abdominal samples for 48 hours.

### 4.7. CT

After PTA staining, we repositioned the samples into their original post-removal positions by securing the sample boards with Velcro to a large acrylic sheet. As detailed in (Nuzov et al. 2025e), the nerve underwent CT imaging (SOMATOM Definition Flash, SIEMENS, Munich, Germany) with 300 μm slice thickness (along the nerve) and in-plane resolution of 0.16 to 0.56 mm.

### 4.8. MicroCT

We cut the nerve to separate all samples using a fresh razor blade for each cut (Nuzov et al. 2025e). As detailed in (Upadhye et al. 2025a), we conducted microCT imaging at 11.4 μm isotropic resolution (SCANCO Medical μCT 100; Wangen-Brüttisellen, Switzerland). We designed a custom cap for the scanner’s tubes to hold two samples placed back-to-back, thus doubling the imaging throughput. Conversely, although the scanner can hold 12 tubes, we only loaded 2 tubes (i.e., four 9-cm samples) at a time to reduce tissue degradation from air exposure and sitting at room temperature.

As detailed in (Zhang et al. 2025), we used 3D Slicer (v5.6.1) (Fedorov et al. 2012) to generate manual segmentations of 100 microCT sub-volumes (each 64 × 1536 × 3072 voxels) from 5 subjects (2 female, 3 male) to train a 3D U-Net for automated segmentation of the fascicle and epineurium boundaries at the native image resolution. We used the Vascular Modeling Toolkit in 3D Slicer (Izzo et al. 2018) to extract the centerlines of nerve and branches from the epineurium segmentations, which we annotated using standardized anatomical terms (Supplement 2) to label the branches and anatomical levels.

### 4.9. Histology and Immunohistochemistry

As detailed in (Coleman et al. 2025), we conducted histological staining and immunohistochemistry (IHC) at select cross sections along the VN. We post-fixed the nerve in 10% formalin for 1 to 4 days. We dissolved the superglue securing each sample to its acrylic board using small amounts of acetone. We grossed each trunk and branch into segments, typically 10–15 mm in length; we then cut each segment into 5 mm-long pieces to embed in one paraffin block, such that a single slice typically yielded two or three nerve cross sections. We carefully documented the grossing process with photos at each step and tissue dye on each piece of tissue to mark orientation. Each paraffin block had a unique label using the subject and sample identifiers, as well as an identifier corresponding to the location of the nerve segment on the gridded acrylic board, as detailed in (Pelot et al. 2025a).

We used an automated tissue processor (HistoCore PEGASUS Tissue Processor, Leica Biosystems, Deer Park, IL, USA) and paraffin embedding station (Leica EG1160). We then took 4 μm slices (Leica AUTOCUT microtome), prioritizing certain anatomical locations, including the typical mid-cervical vagus nerve stimulation (VNS) implant location. We tracked and recorded the slice depth to enable co-registration with microCT. We mounted one slice (with 1 to 3 cross sections) per slide.

Each slide was either stained with hematoxylin and eosin (H&E) or labeled with IHC. We automated H&E staining and coverslipping with the HistoCore SPECTRA Workstation (Leica Biosystems). We automated the IHC processes with the HistoCore SPECTRA Workstation for deparaffinization, the PT Link (Agilent Dako, Santa Clara, CA, USA) for antigen retrieval, and the Autostainer Link 48 (Agilent Dako) for labeling. We developed three IHC protocols: dual labeling with antibodies against myelin basic protein (MBP; 1:4000; MAB20219, Abnova, Taipei City, Taiwan) and neurofilament (NF; 1:2500; MO22103, Neuromics, Minneapolis, MN, USA); single labeling with an antibody against tyrosine hydroxylase (TH; 1:500; MAB318, MilliporeSigma, St. Louis, MO, USA); and single labeling with an antibody against calcitonin gene-related peptide (CGRP; 1:100; GTX82726, GeneTex, Irvine, CA, USA).

We automated imaging with the Axioscan Z7 digital slide scanner (ZEISS, Oberkochen, Germany). We imaged the H&E slides at 20x and the IHC slides at 40x. For the IHC slides, we imaged at multiple focal planes (5 for H&E, 10 for MBP/NF, and 7 for TH and CGRP); we combined the z-stack of images using the “Extended Depth of Focus” option in Zeiss ZEN Microscopy Software to help ensure that the individual neurons were in focus.

We automatically segmented the inner perineurium, outer perineurium, and epineurium boundaries from each H&E image using a 2D nnU-Net (Isensee et al. 2021), and we segmented the fibers identified with IHC with a CellPose model (Stringer et al. 2021).

### 4.10. 3D-MUSE

The VN was processed for 3D-MUSE imaging using a whole-mount staining and embedding protocol (Kolluru et al. 2022). Briefly, we grossed each VN into 15 mm-long segments using a new scalpel blade for each cut. We whole-mount stained with Ehrlich’s hematoxylin to enhance absorptive contrast of nerve fibers and nuclei, followed by graded ethanol dehydration. We added rhodamine B—a fluorescent dye—to the final dehydration step to complement the hematoxylin staining by providing visualization of the nerve’s overall morphology. We infiltrated and embedded the nerve in glycol methacrylate (GMA). We used nerves for 3D-MUSE following excision from the cadaver rather than after microCT because the PTA staining required for CT and microCT interfered with 3D-MUSE image quality.

We mounted the GMA block on the 3D-MUSE system, which combines an ultraviolet (UV) light source, a fluorescence microscope with a motorized stage, and a microtome. We illuminated the block surface obliquely with 280 nm UV light (M280L4; Thorlabs, Newton, NJ, USA). We imaged the block surface using a custom microscope setup incorporating a low-magnification Nikon objective (4×/0.13 NA; N4X-PF - 4X Nikon Plan Fluorite Imaging Objective; Nikon Inc., Melville, NY, USA), a tube lens (TTL-100-A; Thorlabs), and a grayscale camera (Blackfly S BFS-U3–120S4M; FLIR, Wilsonville, OR, USA), providing a field of view of ~3.6 mm × 2.7 mm per tile (0.9 μm × 0.9 μm pixels). The imaging system is mounted on a three-axis motorized stage (Zaber, Vancouver, Canada) for precise automated focusing and tiling. We used a tungsten carbide blade to remove a 3 μm slice before repeating imaging. We used Micro-Manager—an open-source microscope control software—to manage the serial sectioning and image acquisition.

We developed a custom Python preprocessing pipeline, including stitching, contrast enhancement, cropping, and conversion to Zarr files. To segment the morphology (inner perineurium, outer perineurium, and nerve boundaries), we first manually segmented one cross section every 60 μm along the nerve; for Figure 3e, this corresponded to 50 images for the 3 mm nerve segment with 3 μm slices. We used those manual segmentations to train a 2D U-Net, which we then applied to all 1000 images. To generate a corresponding tractogram, we defined random seed points within the fascicles of the first image and applied the structure tensor algorithm from NerveTracker (Kolluru et al. 2024).

### 4.11. Co-Registration

We co-registered data across imaging modalities using metadata and coordinates (Figure 1a), rather than the images or segmentations. We co-registered the 3D tracing to the MRI and CT using anatomical landmarks with a rigid transformation (Figure 2g). We co-registered the 3D tracing to the microCT by matching bony anatomical levels (Supplement 3) and mapping locations between levels based on the percentage of superior-inferior distance. For each histology image, we estimated the corresponding microCT cross section based on histology metadata, from coarse to fine levels of detail (Figure 1; Figure 3a, b): sample, grid coordinate, slice depth, and scene (i.e., the specific nerve cross section within a given slice). For each IHC image, we identified the nearest H&E image based on the histology metadata and calculated the distance between the two cross sections (Figure 3b).

We refined the metadata-based microCT and histology co-registration using the fascicle segmentations (Figure 3d). MBP fascicle segmentations were generated using a grayscale intensity threshold with morphological cleaning and manual validation. We preprocessed the segmented fascicle masks from microCT, H&E, and IHC by aligning the centers of mass, cropping or padding to achieve a bounding box around the tissue region with a 15% margin, and resampling to a common 3 μm isotropic resolution. We co-registered the fascicle segmentations using the SimpleITK framework v2.5.2 (Lowekamp et al. 2013) in Python v3.10. In the rigid alignment stage, we performed Euler (IHC to H&E) or similarity (H&E to microCT) transforms with center-of-mass initialization. We optimized the Euler and similarity transforms on binary masks using mean squares metric and regular step gradient descent with a learning rate of 1.0, minimum step size of 0.01, maximum of 100 iterations, relaxation factor of 0.5, and optimizer scales of 10.0, 1.0, 0.001, and 0.001 for scale, rotation, and translation parameters respectively. In the non-rigid refinement stage, we performed a cubic B-spline transform (order = 3) with an 8×8 control point grid initialized with the previously calculated Euler or similarity transform and identity B-spline. We optimized the B-spline transform on binary masks using the L-BFGS-B algorithm with gradient convergence tolerance of 1×10^−5^, maximum of 100 iterations, 5 corrections, maximum of 1024 function evaluations, and cost function convergence factor of 1×10^7^. We applied the resulting transforms to the centroids of the IHC fiber segmentations to define the fiber coordinates in H&E space and in microCT space.

We stored co-registration information in JSON files: for cadaver CT, MRI, 3D tracing, and histology, each subject has a JSON file, and for microCT, each sample has a JSON file. Each JSON includes the following fields:

1. Name of imaging modality
2. Subject identifier
3. For microCT and histology: sample identifier
4. For histology:
  - “Block” identifier: coordinates on the gridded acrylic boards for the paraffin block
  - “Scene” identifier, to account for multiple nerve cross sections from a given slice c. Slice depth
  - Calculated slice depth per sample and per region (cervical or thoracic)
  - Name of stain or primary antibody
5. Information on the modality’s resolution and coordinate system orientation
  - Number of dimensions
  - Order of anatomical axes: how the axes of the image array correspond to physical, anatomical directions within the subject being imaged (e.g., RAS (right, anterior, superior) or LPI (left, posterior, inferior))
  - Order of image axes: how the dimensions of the image data are indexed within the array (e.g., ZYX, XY)
  - Pixel count and size along each image axis
6. Co-registration data
  - CT
    - Anatomical landmarks
  - MRI
    - Anatomical landmarks
  - 3D tracing
    - Anatomical landmarks shared with MRI
    - Anatomical landmarks shared with CT
    - Anatomical levels
    - Branch points (i.e., the most proximal point of a branch)
  - MicroCT
    - Horizontal grid lines on gridded acrylic boards
    - Centerline xyz coordinates of each anatomical level present for that sample
    - Translation matrices between each sample (i.e., grid)
  - Histology
    - Anatomical label (using the standardized term list, Supplement 2) corresponding to the name of the branch, non-vagal nerve, or anatomical level; if none of those applied, the label indicated the anatomical region of the vagal trunk (right/left/anterior/posterior + cervical/thoracic/esophageal/abdominal)
    - Approximate corresponding microCT image (sample identifier + z-slice), where the microCT z-slice was calculated by the histology “block” coordinates and the microCT gridline annotations

### 4.12. Computational Modeling

We implemented anatomically realistic computational models of human VNS using ASCENT v1.4.0 with Python v3.11.13, COMSOL v6.2, PyFibers v0.4.0, and NEURON v8.2.7 (Hines and Carnevale 1997; Musselman et al. 2021; Marshall et al. 2025a). We implemented two three-dimensional finite element models of VNS, using nerve morphologies from segmented H&E images (Figure 4b): left and right mid-cervical (level of the laryngeal prominence) from subject SR042. We inflated each nerve cross section to compensate for 20% shrinkage that occurs during fixation and histological processing (Hursh 1939; Friede and Samorajski 1967; Stickland 1975; Boyd and Kalu 1979). We deformed each nerve to a circle while maintaining the cross-sectional area and displacing the fascicles as needed to mimic the reshaping that occurs with chronic cuff implantation (Tyler and Durand 2003), enforcing ≥10 μm between adjacent fascicles and from each fascicle to the nerve boundary.

We extruded each deformed nerve cross section to a length of 50 mm. We instrumented each nerve with the standard clinical bipolar helical VNS cuff electrode (3 mm inner diameter) (Musselman et al. 2023), placed halfway along the nerve length, with a 100 μm layer of saline between the nerve and the inner surface of the electrode. We placed the nerve and cuff electrode in a cylindrical surrounding medium (10 mm diameter, 50 mm length; modeled as muscle). We meshed the nerve with a swept mesh and the other regions with free tetrahedral elements, yielding 9,622,781 and 8,093,909 elements for the left and right models, respectively. We used model dimensions and mesh parameters defined in a prior convergence study of validated models of human VNS (Musselman et al. 2023).

We assigned established electrical conductivity values to the tissues and materials (Table 4). We placed a point source of current in each electrode contact and grounded the outer boundaries of the model (Pelot et al. 2018). We used second order geometry and solution shape functions. For each contact delivering 1 mA of current and the other contact set to 0 mA, we computed electric potentials by solving Laplace’s equation.

We sampled potentials every 1 μm along the centroid of each fascicle because activation thresholds vary little within a fascicle for a given fiber diameter (Davis et al. 2023). We interpolated the potentials to correspond to the compartment spacings of the McIntyre-Richardson-Grill (MRG) double-cable model of myelinated axons. We longitudinally aligned the middle node of Ranvier with the longitudinal center of the nerve. We simulated MRG fibers from 2 to 16 μm in diameter, in 2 μm increments with a 1 μs time step, backwards Euler integration, and 50 ms simulation time. We stimulated each fiber with a symmetric biphasic waveform (250 μs/phase; 1 ms start time)—superposing the extracellular potentials from the two contacts. We determined activation thresholds using a bisection search, terminating when the upper and lower search bounds were with 1% of each other. We detected a propagating action potential when the transmembrane potential crossed −30 mV with a rising edge at the node of Ranvier closest to 90% of the fiber length. We interpolated the resulting thresholds in each fascicle across fiber diameters using a quadratic function. We mapped the interpolated thresholds to the fiber diameters and locations from the segmented myelinated fibers from the MBP/NF immunohistochemistry image, co-registered to the H&E-based morphology (Figure 4b). We excluded fibers that were >16 μm (3) or <2 μm (500).

## Supplementary Material

Supplement 1

## Figures and Tables

**Table 1.** Data types and sizes across imaging modalities.

Data collection type	Primary data content and file formats	Approximate data size per cadaver (GB)
CT (pre-dissection)	• 3D imaging data (DICOM, compressed NifTI [Note 1])	4.3–17.9
MRI (pre-dissection)	• Imaging parameters (PDF) • 3D imaging data (DiCoM, compressed NifTI [Note 1])	1.3–4.5
Photos and measurements during dissection	• Dissection notes (PDF) • Branching patterns and nerve diameters (XLSX) • *In situ* photos (JPG) • Labeled photos of excised nerve (PDF)	2–4.5
3D tracing	• xyz coordinates of the vagus nerve, other nearby nerves, anatomical levels, and anatomical landmarks (CSV, OBJ)	0.06–0.2
CT (excised nerve)	• 3D imaging data (DICOM, compressed NifTI [Note 1])	1.4–5.8
MicroCT	• 3D imaging data (compressed Zarr [Note 2], OME-TIFF [Note 3]) • Centerlines of the vagus nerve, centerlines of other nerves, and points defining anatomical levels (CSV) • Accessible visualizations (PNG, MP4, PDF)	550–650
Histology	• 2D imaging data (OME-TIFF) • Accessible visualizations (PDF)	44–1170
3D-MUSE	• 3D imaging data (compressed Zarr [Note 2]) • Accessible visualizations (PDF)	10 [Note 4]

[Note 1] .nii.gz. [Note 2] .zarr.zip. [Note 3] Downsampled 4x. [Note 4] Per 15 mm length of nerve, rather than per cadaver.

**Table 2.** Methods published on protocols.io for dissection, tissue handling, and multi-modal imaging of cadaveric human vagus nerves.

Protocol title	Entry on protocols.io
REVA #1: Cadaver Embalming and Preparation for Whole Body Imaging	(Nuzov et al. 2025a)
REVA #2: Computed Tomography (CT) of Embalmed Cadaver	(Nuzov et al. 2025d)
REVA #3: Magnetic Resonance Imaging (MRI) of Embalmed Cadaver	(Herzka et al. 2025)
REVA #4: Labeling of Vagus Nerve Subjects, Samples, and Paraffin Blocks	(Pelot et al. 2025a)
REVA #5: Dissecting and Measuring Cadaveric Human Vagus Nerves	(Brunsman et al. 2025)
REVA #6: 3D Tracing of Cadaveric Human Vagus Nerves	(Nuzov et al. 2025b)
REVA #7: Excision of Cadaveric Human Vagus Nerves	(Nuzov et al. 2025c)
REVA #8: Phosphotungstic Acid (PTA) Staining of Cadaveric Human Vagus Nerves	(Upadhye et al. 2025b)
REVA #9: Computed Tomography (CT) of Excised Cadaveric Human Vagus Nerves	(Nuzov et al. 2025e)
REVA #10: Microcomputed Tomography (MicroCT) of Excised Cadaveric Human Vagus Nerves	(Upadhye et al. 2025a)
REVA #11: Histology and Immunohistochemistry (IHC) of Cadaveric Human Vagus Nerves	(Coleman et al. 2025)

**Table 3:** MRI pulse sequence parameters.

	CISS*	Dixon-VIBE	VIBE
**Orientation**	Sagittal	Axial	Sagittal
**Minimum TR**	5.99 ms	10.00 ms	10.00 ms
**TE**	3.00 ms	Echo 1: 2.46 ms Echo 2: 3.69 ms	2.46 ms
**Flip angle**	35 deg	10 deg	12 deg
**FOVx**	256 mm	405 mm	323 mm
**FOVy** **	256 mm	278 mm	212 mm
**FOVz** **	224 mm	145.6 mm	128 mm
**Matrix (X, Y)** **	512, 512	576, 396	640, 420
**Number of slices**	448	208	256
**Oversampling in z**	0.0%	15.4%	12.5%
**Oversampling in y**	0.0%	10%	10%
**Elliptical scanning window**	on	off	on
**Scan duration per station**	35 min 57 sec	10 min 28 sec	14 min 23 sec
**Number of stations** ***	4	7	3
**Bandwidth**	488 Hz/Px	430 Hz/Px	240 Hz/Px
**Averages**	1	1	1
**Orientation**	sagittal	axial	sagittal
**Voxel size** ****	Acquired: 0.5 mm isotropic Reconstructed: 0.5 mm isotropic	Acquired: 0.7 mm isotropic Reconstructed: 0.7 mm isotropic	Acquired: 0.5 mm * 0.6 mm * 0.6 mm Reconstructed: 0.5 mm isotropic
**Dimensionality (2D vs 3D)**	3D	3D	3D

* Modified pulse sequence was used to achieve required coverage.

** Adapted for full anterior-posterior coverage dependent on body habitus; resolution was fixed at 0.5 mm.

*** Adapted depending on the height of the subject.

**** 6/8 partial Fourier undersampling in both slice and phase for Dixon-VIBE.

**Table 4.** Material and tissue conductivities for computational models of human vagus nerve stimulation.

Material or tissue	Electrical conductivity (S/m)	References
Endoneurium	{0.167, 0.167, 0.571}	(Ranck and Bement 1965; Pelot et al. 2019)
Perineurium	0.000 8703	(Weerasuriya et al. 1984; Pelot et al. 2019)
Epineurium	0.159	(Grill and Mortimer 1994; Stolinski 1995; Pelot et al. 2017)
Saline	1.76	(Horch and Kipke 2017)
Platinum	9.43 × 10^6^	(De Podesta et al. 1996)
Silicone	10^−12^	(Callister and Rethwisch 2013)
Muscle	{0.086, 0.086, 0.35}	(Gielen et al. 1984)

## Data Availability

The data for each modality will be made publicly available for an example subject on sparc.science upon paper acceptance.

## Footnotes

1 https://zarr.dev/ (accessed April 19, 2025)

2 https://docs.openmicroscopy.org/ome-model/5.6.3/ome-tiff/ (accessed April 19, 2025)

3 https://www.globus.org/ (accessed April 19, 2025)

4 https://discover.pennsieve.io/ (accessed April 19, 2025)

5 https://sparc.science/ (accessed April 19, 2025)

6 https://about.gitlab.com/ (accessed April 19, 2025)

## References

[R1] Anand IS, Konstam MA, Klein HU, Mann DL, Ardell JL, Gregory DD, Comparison of symptomatic and functional responses to vagus nerve stimulation in ANTHEM-HF, INOVATE-HF, and NECTAR-HF. ESC Heart Fail. 2020 Feb;7(1):75–83. 31984682 10.1002/ehf2.12592 PMC7083506

[R2] Ardell JL, Rajendran PS, Nier HA, KenKnight BH, Armour JA. Central-peripheral neural network interactions evoked by vagus nerve stimulation: functional consequences on control of cardiac function. Am J Physiol Heart Circ Physiol. 2015 Nov 15;309(10):H1740–1752. 26371171 10.1152/ajpheart.00557.2015 PMC4666982

[R3] Aristovich K, Donega M, Fjordbakk C, Tarotin I, Chapman CAR, Viscasillas J, Model-based geometrical optimisation and in vivo validation of a spatially selective multielectrode cuff array for vagus nerve neuromodulation. J Neurosci Methods. 2021 Mar 15;352:109079. 33516735 10.1016/j.jneumeth.2021.109079

[R4] Austelle CW, Cox SS, Wills KE, Badran BW. Vagus nerve stimulation (VNS): recent advances and future directions. Clin Auton Res. 2024 Dec;34(6):529–47. 39363044 10.1007/s10286-024-01065-w PMC11543756

[R5] Bandrowski A, Grethe JS, Pilko A, Gillespie T, Pine G, Patel B, SPARC Data Structure: Rationale and Design of a FAIR Standard for Biomedical Research Data [Internet]. Bioinformatics; 2021 Feb. Available from: http://biorxiv.org/lookup/doi/10.1101/2021.02.10.430563

[R6] Berthoud HR, Neuhuber WL. Functional and chemical anatomy of the afferent vagal system. Auton Neurosci. 2000 Dec 20;85(1–3):1–17. 11189015 10.1016/S1566-0702(00)00215-0

[R7] Bonaz B, Sinniger V, Pellissier S. Vagus nerve stimulation: a new promising therapeutic tool in inflammatory bowel disease. J Intern Med. 2017 Jul;282(1):46–63. 28421634 10.1111/joim.12611

[R8] Boyd IA, Kalu KU. Scaling factor relating conduction velocity and diameter for myelinated afferent nerve fibres in the cat hind limb. J Physiol-London. 1979 Apr;289:277–97. 458657 10.1113/jphysiol.1979.sp012737 PMC1281370

[R9] Breit S, Kupferberg A, Rogler G, Hasler G. Vagus Nerve as Modulator of the Brain-Gut Axis in Psychiatric and Inflammatory Disorders. Front Psychiatry. 2018;9:44. 29593576 10.3389/fpsyt.2018.00044 PMC5859128

[R10] Brunsman B, Lunasco L, Workman K, Nuzov N, Bokhari S, Pascol T, REVA #5: Dissecting and Measuring Cadaveric Human Vagus Nerves v1 [Internet]. 2025 [cited 2025 Sep 9]. Available from: https://www.protocols.io/view/reva-5-dissecting-and-measuring-cadaveric-human-va-g8skbzwcx

[R11] Bucksot JE, Wells AJ, Rahebi KC, Sivaji V, Romero-Ortega M, Kilgard MP, Flat electrode contacts for vagus nerve stimulation. Nógrádi A, editor. PLoS ONE. 2019 Nov 18;14(11):e0215191. 31738766 10.1371/journal.pone.0215191 PMC6862926

[R12] Bydder GM, Kreel L. The temperature dependence of computed tomography attenuation values. J Comput Assist Tomogr. 1979 Aug;3(4):506–10. 457964 10.1097/00004728-197908000-00013

[R13] Callister WD, Rethwisch DG. Fundamentals of materials science and engineering. 4. ed., SI version. Hoboken, N.J: Wiley; 2013.

[R14] Coleman J, Cintron E, Kim Y, Hartzler A, Islam P, Jeter T, REVA #11: Histology and Immunohistochemistry (IHC) of Cadaveric Human Vagus Nerves v1 [Internet]. 2025 [cited 2025 Sep 9]. Available from: https://www.protocols.io/view/reva-11-histology-and-immunohistochemistry-ihc-of-g8stbzwep

[R15] Couppey T, Regnacq L, Giraud R, Romain O, Bornat Y, Kolbl F. NRV: An open framework for in silico evaluation of peripheral nerve electrical stimulation strategies. Schwab BC, editor. PLoS Comput Biol. 2024 Jul 12;20(7):e1011826. 38995970 10.1371/journal.pcbi.1011826 PMC11268605

[R16] Dali M, William L, Tigra W, Taillades H, Rossel O, Azevedo C, Relevance of selective neural stimulation with a multicontact cuff electrode using multicriteria analysis. Cymbalyuk G, editor. PLoS ONE. 2019 Jul 2;14(7):e0219079. 31265480 10.1371/journal.pone.0219079 PMC6605667

[R17] Davis CJ, Musselman ED, Grill WM, Pelot NA. Fibers in smaller fascicles have lower activation thresholds with cuff electrodes due to thinner perineurium and smaller cross-sectional area. J Neural Eng [Internet]. 2023 Mar 14 [cited 2023 Mar 17]; Available from: https://iopscience.iop.org/article/10.1088/1741-2552/acc42b 10.1088/1741-2552/acc42b PMC10410695 36917856

[R18] Dawson J, Engineer ND, Prudente CN, Pierce D, Francisco G, Yozbatiran N, Vagus Nerve Stimulation Paired With Upper-Limb Rehabilitation After Stroke: One-Year Follow-up. Neurorehabil Neural Repair. 2020 Jun 1;154596832092436. 10.1177/1545968320924361 32476617

[R19] De Podesta M, Laboratory NP, Uk. Understanding the Properties of Matter [Internet]. Abingdon, UK: Taylor & Francis; 1996 [cited 2025 Aug 24]. Available from: https://www.taylorfrancis.com/books/9780203450611

[R20] Doubilet H, Shafiroff BG, Mulholland JH. The Anatomy of the Peri-Esophageal Vagi. Ann Surg. 1948 Jan;127(1):128–35. 17859054 10.1097/00000658-194801000-00012 PMC1513771

[R21] Eiber CD, Payne SC, Biscola NP, Havton LA, Keast JR, Osborne PB, Computational modelling of nerve stimulation and recording with peripheral visceral neural interfaces. J Neural Eng. 2021 Nov 25;18(6). 10.1088/1741-2552/ac36e2 PMC8903799 34740201

[R22] Farrand AQ, Verner RS, McGuire RM, Helke KL, Hinson VK, Boger HA. Differential effects of vagus nerve stimulation paradigms guide clinical development for Parkinson’s disease. Brain Stimulation. 2020 Sep;13(5):1323–32. 32629028 10.1016/j.brs.2020.06.078

[R23] Fereidouni F, Harmany ZT, Tian M, Todd A, Kintner JA, McPherson JD, Microscopy with ultraviolet surface excitation for rapid slide-free histology. Nat Biomed Eng. 2017 Dec;1(12):957–66. 31015706 10.1038/s41551-017-0165-y PMC6223324

[R24] Fisher LE, Tyler DJ, Triolo RJ. Optimization of selective stimulation parameters for multi-contact electrodes. J NeuroEngineering Rehabil. 2013;10(1):25. 10.1186/1743-0003-10-25 PMC3599334 23442372

[R25] Fitchett A, Mastitskaya S, Aristovich K. Selective Neuromodulation of the Vagus Nerve. Front Neurosci. 2021 May 24;15:685872. 34108861 10.3389/fnins.2021.685872 PMC8180849

[R26] Friede RL, Samorajski T. Relation between the number of myelin lamellae and axon circumference in fibers of vagus and sciatic nerves of mice. J Comp Neurol. 1967 Jul;130(3):223–31. 6036111 10.1002/cne.901300304

[R27] Gielen FL, Wallinga-de Jonge W, Boon KL. Electrical conductivity of skeletal muscle tissue: experimental results from different muscles in vivo. Med Biol Eng Comput. 1984 Nov;22(6):569–77. 6503387 10.1007/BF02443872

[R28] Grill WM, Mortimer JT. Electrical properties of implant encapsulation tissue. Ann Biomed Eng. 1994 Jan;22(1):23–33. 8060024 10.1007/BF02368219

[R29] Hammer N, Glätzner J, Feja C, Kühne C, Meixensberger J, Planitzer U, Human Vagus Nerve Branching in the Cervical Region. Tache Y, editor. PLOS ONE. 2015 Feb 13;10(2):e0118006. 25679804 10.1371/journal.pone.0118006 PMC4332499

[R30] Handforth A, DeGiorgio CM, Schachter SC, Uthman BM, Naritoku DK, Tecoma ES, Vagus nerve stimulation therapy for partial-onset seizures: a randomized active-control trial. Neurology. 1998 Jul;51(1):48–55. 9674777 10.1212/wnl.51.1.48

[R31] Havton LA, Biscola NP, Stern E, Mihaylov PV, Kubal CA, Wo JM, Human organ donor-derived vagus nerve biopsies allow for well-preserved ultrastructure and high-resolution mapping of myelinated and unmyelinated fibers. Sci Rep. 2021 Dec;11(1):23831. 34903749 10.1038/s41598-021-03248-1 PMC8668909

[R32] Helmers SL, Begnaud J, Cowley A, Corwin HM, Edwards JC, Holder DL, Application of a computational model of vagus nerve stimulation. Acta Neurologica Scandinavica. 2012 Nov;126(5):336–43. 22360378 10.1111/j.1600-0404.2012.01656.x

[R33] Herzka D, Markley M, Nuzov N, Sali G, Kumari S, Pelot N, REVA #3: Magnetic Resonance Imaging (MRI) of Embalmed Cadaver v1 [Internet]. 2025 [cited 2025 Sep 9]. Available from: https://www.protocols.io/view/reva-3-magnetic-resonance-imaging-mri-of-embalmed-g8sibzwcf

[R34] Hines ML, Carnevale NT. The NEURON simulation environment. Neural Comput. 1997 Aug 15;9(6):1179–209. 9248061 10.1162/neco.1997.9.6.1179

[R35] Horch KW, Kipke D, editors. Neuroprosthetics: theory and practice. 2nd edition. New Jersey: World Scientific; 2017. (Series on bioengineering and biomedical engineering).

[R36] Hursh JB. Conduction Velocity and Diameter of Nerve Fibers. American Journal of Physiology. 1939;127(1):131–9.

[R37] Hussain MA, Grill WM, Pelot NA. Highly efficient modeling and optimization of neural fiber responses to electrical stimulation. Nat Commun. 2024 Aug 31;15(1):7597. 39217179 10.1038/s41467-024-51709-8 PMC11365978

[R38] Ikramuddin S, Blackstone RP, Brancatisano A, Toouli J, Shah SN, Wolfe BM, Effect of reversible intermittent intra-abdominal vagal nerve blockade on morbid obesity: the ReCharge randomized clinical trial. JAMA. 2014 Sep 3;312(9):915–22. 25182100 10.1001/jama.2014.10540

[R39] Isensee F, Jaeger PF, Kohl SAA, Petersen J, Maier-Hein KH. nnU-Net: a self-configuring method for deep learning-based biomedical image segmentation. Nat Methods. 2021 Feb;18(2):203–11. 33288961 10.1038/s41592-020-01008-z

[R40] Jabaley ME, Wallace WH, Heckler FR. Internal topography of major nerves of the forearm and hand: a current view. J Hand Surg Am. 1980 Jan;5(1):1–18. 7365209 10.1016/s0363-5023(80)80035-9

[R41] Jayaprakash N, Song W, Toth V, Vardhan A, Levy T, Tomaio J, Organ- and function-specific anatomical organization of vagal fibers supports fascicular vagus nerve stimulation. Brain Stimul. 2023 Feb 10;16(2):484–506. 36773779 10.1016/j.brs.2023.02.003 PMC10228508

[R42] Johnson RL, Wilson CG. A review of vagus nerve stimulation as a therapeutic intervention. J Inflamm Res. 2018;11:203–13. 29844694 10.2147/JIR.S163248 PMC5961632

[R43] Kamoga R, Rukundo GZ, Kalungi S, Adriko W, Nakidde G, Obua C, Vagus nerve stimulation in dementia: A scoping review of clinical and pre-clinical studies. AIMS Neurosci. 2024;11(3):398–420. 39431268 10.3934/Neuroscience.2024024 PMC11486617

[R44] Kaniusas E, Kampusch S, Tittgemeyer M, Panetsos F, Gines RF, Papa M, Current Directions in the Auricular Vagus Nerve Stimulation I - A Physiological Perspective. Front Neurosci. 2019;13:854. 31447643 10.3389/fnins.2019.00854 PMC6697069

[R45] Kawagishi K, Fukushima N, Yokouchi K, Sumitomo N, Kakegawa A, Moriizumi T. Tyrosine hydroxylase-immunoreactive fibers in the human vagus nerve. J Clin Neurosci. 2008 Sep;15(9):1023–6. 18617399 10.1016/j.jocn.2007.08.032

[R46] Kolluru C, Joseph N, Seckler J, Fereidouni F, Levenson R, Shoffstall A, NerveTracker: a Python-based software toolkit for visualizing and tracking groups of nerve fibers in serial block-face microscopy with ultraviolet surface excitation images. J Biomed Opt. 2024 Jul;29(7):076501. 38912214 10.1117/1.JBO.29.7.076501 PMC11188586

[R47] Kolluru C, Todd A, Upadhye AR, Liu Y, Berezin MY, Fereidouni F, Imaging peripheral nerve micro-anatomy with MUSE, 2D and 3D approaches. Sci Rep. 2022 Jun 17;12(1):10205. 35715554 10.1038/s41598-022-14166-1 PMC9205958

[R48] Kronsteiner B, Zopf LM, Heimel P, Oberoi G, Kramer AM, Slezak P, Mapping the functional anatomy and topography of the cardiac autonomic innervation for selective cardiac neuromodulation using MicroCT. Front Cell Dev Biol. 2022;10:968870. 36172280 10.3389/fcell.2022.968870 PMC9511100

[R49] Labuschagne JJ, Hammer N. Duplicated Vagus Nerve in Adolescence: Case Report and Review of Literature. World Neurosurgery. 2019 Nov;131:180–5. 31408750 10.1016/j.wneu.2019.08.014

[R50] Lowekamp BC, Chen DT, Ibáñez L, Blezek D. The Design of SimpleITK. Front Neuroinform [Internet]. 2013 [cited 2025 Sep 15];7. Available from: http://journal.frontiersin.org/article/10.3389/fninf.2013.00045/abstract 10.3389/fninf.2013.00045 PMC3874546 24416015

[R51] Lubba CH, Le Guen Y, Jarvis S, Jones NS, Cork SC, Eftekhar A, PyPNS: Multiscale Simulation of a Peripheral Nerve in Python. Neuroinform. 2019 Jan;17(1):63–81. 10.1007/s12021-018-9383-z PMC6394768 29948844

[R52] Márquez-Rivas J, Garcia-Armengol R, Serrano MT, Blanco-Martínez B, Becerra Cuñat JL, Vázquez-Mateos C, PO097 / #1036 VAGAL NERVE DUPLICATION DURING VNS INTERVENTION: A CASE SERIES. Neuromodulation: Technology at the Neural Interface. 2022 Oct;25(7):S238.

[R53] Marshall DP, Farah ES, Musselman ED, Pelot NA, Grill WM. PyFibers: An open-source NEURON-Python package to simulate responses of model nerve fibers to electrical stimulation. 2025a;. 10.1371/journal.pcbi.1013764 PMC12700385 41385504

[R54] Marshall DP, Upadhye AR, Buyukcelik ON, Shoffstall AJ, Grill WM, Pelot NA. Computational Modeling of Human Vagus Nerve Stimulation with Three-Dimensional Fascicular Morphology. 2025b;. 10.1063/5.0308450 PMC12956375 41782809

[R55] Mizeres NJ. The cardiac plexus in man. Am J Anat. 1963 Mar;112(2):141–51.

[R56] Musselman ED, Cariello JE, Grill WM, Pelot NA. ASCENT (Automated Simulations to Characterize Electrical Nerve Thresholds): A pipeline for sample-specific computational modeling of electrical stimulation of peripheral nerves. PLoS Comput Biol. 2021 Sep;17(9):e1009285. 34492004 10.1371/journal.pcbi.1009285 PMC8423288

[R57] Musselman ED, Pelot NA, Grill WM. Validated computational models predict vagus nerve stimulation thresholds in preclinical animals and humans. J Neural Eng. 2023 Jun 15;20(3). 10.1088/1741-2552/acda64 PMC10324064 37257454

[R58] Musselman ED, Raha I, Pelot NA, Grill WM. Scaling of vagus nerve stimulation parameters does not achieve equivalent nerve responses across species. Bioelectron Med. 2025 May 16;11(1):11. 40375300 10.1186/s42234-025-00174-9 PMC12083175

[R59] Neuhuber WL, Berthoud HR. Functional anatomy of the vagus system – Emphasis on the somato-visceral interface. Autonomic Neuroscience. 2021 Dec;236:102887. 34634680 10.1016/j.autneu.2021.102887 PMC8627476

[R60] Nuzov N, Brunsman B, Pelot N, Shoffstall A, Crofton A. REVA #1: Cadaver Embalming and Preparation for Whole Body Imaging v1 [Internet]. 2025a [cited 2025 Sep 9]. Available from: https://www.protocols.io/view/reva-1-cadaver-embalming-and-preparation-for-whole-g8sgbzwbx

[R61] Nuzov N, Lam V, Pelot N, Crofton A, Shoffstall A. REVA #6: 3D Tracing of Cadaveric Human Vagus Nerves v1 [Internet]. 2025b [cited 2025 Sep 9]. Available from: https://www.protocols.io/view/reva-6-3d-tracing-of-cadaveric-human-vagus-nerves-g8smbzwc7

[R62] Nuzov N, Lunasco L, Brunsman B, Workman K, Lam V, Samba Z, REVA #7: Excision of Cadaveric Human Vagus Nerves v1 [Internet]. 2025c [cited 2025 Sep 9]. Available from: https://www.protocols.io/view/reva-7-excision-of-cadaveric-human-vagus-nerves-g8spbzwdp

[R63] Nuzov N, Pelot N, Shoffstall A. REVA #2: Computed Tomography (CT) of Embalmed Cadaver v1 [Internet]. 2025d [cited 2025 Sep 9]. Available from: https://www.protocols.io/view/reva-2-computed-tomography-ct-of-embalmed-cadaver-g8shbzwb7

[R64] Nuzov N, Pelot N, Shoffstall A. REVA #9: Computed Tomography (CT) of Excised Cadaveric Human Vagus Nerves v1 [Internet]. 2025e [cited 2025 Sep 9]. Available from: https://www.protocols.io/view/reva-9-computed-tomography-ct-of-excised-cadaveric-g8srbzwd7

[R65] Ottaviani MM, Macefield VG. Structure and Functions of the Vagus Nerve in Mammals. Compr Physiol. 2022 Aug 11;12(4):3989–4037. 35950655 10.1002/cphy.c210042

[R66] Pandeya GD, Greuter MJW, Schmidt B, Flohr T, Oudkerk M. Assessment of thermal sensitivity of CT during heating of liver: an ex vivo study. Br J Radiol. 2012 Sep;85(1017):e661–665. 22919016 10.1259/bjr/23942179 PMC3487082

[R67] Pelot N, Nuzov N, Coleman J, Shoffstall A. REVA #4: Labeling of Vagus Nerve Subjects, Samples, and Paraffin Blocks v1 [Internet]. 2025a [cited 2025 Sep 9]. Available from: https://www.protocols.io/view/reva-4-labeling-of-vagus-nerve-subjects-samples-an-g8sjbzwcp

[R68] Pelot NA, Behrend CE, Grill WM. Modeling the response of small myelinated axons in a compound nerve to kilohertz frequency signals. J Neural Eng. 2017;14(4):046022. 28361793 10.1088/1741-2552/aa6a5f PMC5677574

[R69] Pelot NA, Behrend CE, Grill WM. On the parameters used in finite element modeling of compound peripheral nerves. Journal of Neural Engineering. 2019 Feb 1;16(1):016007. 30507555 10.1088/1741-2552/aaeb0c PMC7309635

[R70] Pelot NA, Goldhagen GB, Cariello JE, Musselman ED, Clissold KA, Ezzell JA, Quantified morphology of the cervical and subdiaphragmatic vagus nerves of human, pig, and rat. Front Neurosci. 2020 Nov 4;14:601479. 33250710 10.3389/fnins.2020.601479 PMC7672126

[R71] Pelot NA, Thio BJ, Grill WM. Modeling current sources for neural stimulation in COMSOL. Frontiers in Computational Neuroscience [Internet]. 2018 Jun 8 [cited 2018 Jun 9];12. Available from: https://www.frontiersin.org/article/10.3389/fncom.2018.00040/full 10.3389/fncom.2018.00040 PMC6002501 29937722

[R72] Pelot NA, Wang B, Marshall DP, Hussain MA, Musselman ED, Yu GJ, Guidance for sharing computational models of neural stimulation: from project planning to publication. J Neural Eng [Internet]. 2025b Feb 24 [cited 2025 Mar 1]; Available from: https://iopscience.iop.org/article/10.1088/1741-2552/adb997 10.1088/1741-2552/adb997 PMC12369073 39993327

[R73] Peterson D, Van Poppel M, Boling W, Santos P, Schwalb J, Eisenberg H, Clinical safety and feasibility of a novel implantable neuroimmune modulation device for the treatment of rheumatoid arthritis: initial results from the randomized, double-blind, sham-controlled RESET-RA study. Bioelectron Med. 2024 Mar 13;10(1):8. 38475923 10.1186/s42234-023-00138-x PMC10935935

[R74] Ranck JB Jr, Bement SL. The specific impedance of the dorsal columns of cat: an anisotropic medium. Exp Neurol. 1965 Apr;11:451–63. 14278100 10.1016/0014-4886(65)90059-2

[R75] Ravi N, Gabeur V, Hu YT, Hu R, Ryali C, Ma T, SAM 2: Segment Anything in Images and Videos [Internet]. arXiv; 2024 [cited 2025 Apr 10]. Available from: https://arxiv.org/abs/2408.00714

[R76] Romeni S, Losanno E, Koert E, Pierantoni L, Delgado-Martínez I, Navarro X, Combining biophysical models and machine learning to optimize implant geometry and stimulation protocol for intraneural electrodes. J Neural Eng. 2023 Jun 27; 10.1088/1741-2552/ace219 37369194

[R77] Ronneberger O, Fischer P, Brox T. U-Net: Convolutional Networks for Biomedical Image Segmentation. 2015 [cited 2023 Sep 17]; Available from: https://arxiv.org/abs/1505.04597

[R78] Ruder TD, Hatch GM, Siegenthaler L, Ampanozi G, Mathier S, Thali MJ, The influence of body temperature on image contrast in post mortem MRI. European Journal of Radiology. 2012 Jun;81(6):1366–70. 21458188 10.1016/j.ejrad.2011.02.062

[R79] Rush AJ, Marangell LB, Sackeim HA, George MS, Brannan SK, Davis SM, Vagus nerve stimulation for treatment-resistant depression: a randomized, controlled acute phase trial. Biol Psychiatry. 2005 Sep 1;58(5):347–54. 16139580 10.1016/j.biopsych.2005.05.025

[R80] Schnitzlein HN, Rowe LC, Hoffman HH. The myelinated component of the vagus nerves in man. The Anatomical Record. 1958 Aug;131(4):649–67.

[R81] Schulz G, Crooijmans HJA, Germann M, Scheffler K, Müller-Gerbl M, Müller B. Three-dimensional strain fields in human brain resulting from formalin fixation. J Neurosci Methods. 2011 Oct 30;202(1):17–27. 21889536 10.1016/j.jneumeth.2011.08.031

[R82] Seki A, Green HR, Lee TD, Hong L, Tan J, Vinters HV, Sympathetic nerve fibers in human cervical and thoracic vagus nerves. Heart Rhythm. 2014 Aug;11(8):1411–7. 24768897 10.1016/j.hrthm.2014.04.032 PMC4108556

[R83] Settell ML, Pelot NA, Knudsen BE, Dingle AM, McConico AL, Nicolai EN, Functional vagotopy in the cervical vagus nerve of the domestic pig: Implications for the study of vagus nerve stimulation. J Neural Eng [Internet]. 2020 Feb 27 [cited 2020 Mar 4]; Available from: https://iopscience.iop.org/article/10.1088/1741-2552/ab7ad4 10.1088/1741-2552/ab7ad4 PMC7306215 32108590

[R84] Stickland NC. A detailed analysis of the effects of various fixatives on animal tissue with particular reference to muscle tissue. Stain Technol. 1975 Jul;50(4):255–64. 810925 10.3109/10520297509117068

[R85] Stolinski C. Structure and composition of the outer connective tissue sheaths of peripheral nerve. J Anat. 1995 Feb;186 (Pt 1):123–30. 7649808 PMC1167278

[R86] Stringer C, Wang T, Michaelos M, Pachitariu M. Cellpose: a generalist algorithm for cellular segmentation. Nat Methods. 2021 Jan;18(1):100–6. 33318659 10.1038/s41592-020-01018-x

[R87] Taschuk M, Wilson G. Ten simple rules for making research software more robust. PLoS Comput Biol. 2017;13(4):e1005412. 28407023 10.1371/journal.pcbi.1005412 PMC5390961

[R88] Thompson N, Ravagli E, Mastitskaya S, Challita R, Hadaya J, Iacoviello F, Towards spatially selective efferent neuromodulation: anatomical and functional organization of cardiac fibres in the porcine cervical vagus nerve. J Physiol. 2025 Mar;603(7):1983–2004. 39183636 10.1113/JP286494 PMC11955868

[R89] Tyler DJ, Durand DM. Chronic Response of the Rat Sciatic Nerve to the Flat Interface Nerve Electrode. Annals of Biomedical Engineering. 2003 Jun;31(6):633–42. 12797612 10.1114/1.1569263

[R90] Upadhye A, Nuzov N, Tsipsis C, Zhang J, Shunmugavel A, Chin J, REVA #10: Microcomputed Tomography (MicroCT) of Excised Cadaveric Human Vagus Nerves v1 [Internet]. 2025a [cited 2025 Sep 9]. Available from: https://www.protocols.io/view/reva-10-microcomputed-tomography-microct-of-excise-g8ssbzwef

[R91] Upadhye A, Zhang J, Nuzov N, Shunmugavel A, Tsipsis C, Chin J, REVA #8: Phosphotungstic Acid (PTA) Staining of Cadaveric Human Vagus Nerves v1 [Internet]. 2025b [cited 2025 Sep 9]. Available from: https://www.protocols.io/view/reva-8-phosphotungstic-acid-pta-staining-of-cadave-g8sqbzwdx

[R92] Upadhye AR, Cintron E, Zhang J, Coleman J, Kolluru C, Jenkins MW, Phosphotungstic Acid Staining to Visualize the Vagus Nerve Perineurium Using Micro-CT. J Neuroimaging. 2025c;35(2):e70040. 40207700 10.1111/jon.70040 PMC11984074

[R93] Upadhye AR, Kolluru C, Druschel L, Al Lababidi L, Ahmad SS, Menendez DM, Fascicles split or merge every ~560 microns within the human cervical vagus nerve. J Neural Eng. 2022 Sep 29; 10.1088/1741-2552/ac9643 PMC10353574 36174538

[R94] Weerasuriya A, Spangler RA, Rapoport SI, Taylor RE. AC impedance of the perineurium of the frog sciatic nerve. Biophys J. 1984 Aug;46(2):167–74. 6332648 10.1016/S0006-3495(84)84009-6 PMC1435024

[R95] Wilson G, Aruliah DA, Brown CT, Chue Hong NP, Davis M, Guy RT, Best practices for scientific computing. PLoS Biol. 2014 Jan;12(1):e1001745. 24415924 10.1371/journal.pbio.1001745 PMC3886731

[R96] Wilson G, Bryan J, Cranston K, Kitzes J, Nederbragt L, Teal TK. Good enough practices in scientific computing. PLoS Comput Biol. 2017 Jun;13(6):e1005510. 28640806 10.1371/journal.pcbi.1005510 PMC5480810

[R97] Wongsarnpigoon A, Grill WM. Energy-efficient waveform shapes for neural stimulation revealed with a genetic algorithm. Journal of Neural Engineering. 2010 Aug 1;7(4):046009. 20571186 10.1088/1741-2560/7/4/046009 PMC2925408

[R98] Zacharewski N, Killory BD. Duplicate Left-Sided Vagus Nerve: Intraoperative Imaging, Management, and Placement. J Neurol Surg Rep. 2023 Apr;84(02):e59–60. 37213414 10.1055/s-0043-1768713 PMC10195161

[R99] Zhang J, Lapierre-Landry M, Kalpatthi H, Jenkins MW, Wilson DL, Pelot NA, Automated 3D Segmentation of Human Vagus Nerve Fascicles and Epineurium from Micro-Computed Tomography Images Using Anatomy-Aware Neural Networks [Internet]. Neuroscience; 2025 [cited 2025 Jun 16]. Available from: http://biorxiv.org/lookup/doi/10.1101/2025.06.12.659370 10.1088/1741-2552/ae33f6 PMC13007042 41494208

