# Supplementary material for: The human vagal complex: from gross anatomy to single neurons, from brainstem to abdomen
Source: Res Sq. 2025 Oct 20:rs.3.rs-7707775. Preprint. [Version 1] doi: 10.21203/rs.3.rs-7707775/v1 (PMC12633527; doi:10.21203/rs.3.rs-7707775/v1)
Supplement: Supplement 1 [file NIHPPRS7707775V1-supplement-1.pdf]

Supplement 1

Cadaver Demographics

Table 5. Cadaver demographics.

Subject identifier	Sex	Race (self-identified)	Age (years)
SR001	Female	White	82
SR002	Male	White	32
SR003	Male	White	67
SR004	Female	Black or African American	87
SR005	Male	Black or African American	77
SR006	Female	White	83
SR007	Male	White	80
SR008	Female	White	79
SR009	Male	Hispanic	57
SR010	Female	White	81
SR011	Male	White	76
SR012	Male	White	77
SR013	Male	White	>89
SR014	Male	White	64
SR015	Female	Black or African American	>89
SR016	Female	White	>89
SR017	Female	Black or African American	>89
SR018	Female	Black or African American	55
SR019	Female	White	78
SR020	Male	White	>89
SR021	Female	White	58
SR022	Male	White	68
SR023	Male	White	82
SR024	Female	Black or African American	89
SR025	Male	White	58
SR026	Female	White	>89
SR027	Male	White	72
SR028	Female	White	>89
SR029	Male	Asian	>89
SR030	Male	Black or African American	41
SR031	Male	Black or African American	76
SR032	Female	White	>89
SR033	Female	White	89
SR034	Male	White	82
SR035	Female	White	85
SR036	Female	White	82
SR037	Female	White	76
SR038	Female	White	67
SR039	Female	Black or African American	82
SR040	Female	White	>89
SR041	Male	White	67
SR042	Female	Black or African American	36
SR043	Female	White	>89
SR044	Female	White	81
SR045	Female	White	>89
SR046	Male	White	86
SR047	Male	White	71
SR048	Male	White	48
SR049	Female	White	75
SR050	Male	White	54
SR051	Female	Black or African American	49
SR052	Male	White	53
SR053	Male	White	38
SR054	Male	White	81
SR055	Male	White	74
SR056	Female	White	>89
SR057	Female	Black or African American; Hispanic	63
SR058	Female	White	>89
SR059	Female	Black or African American	88
SR060	Female	White	61

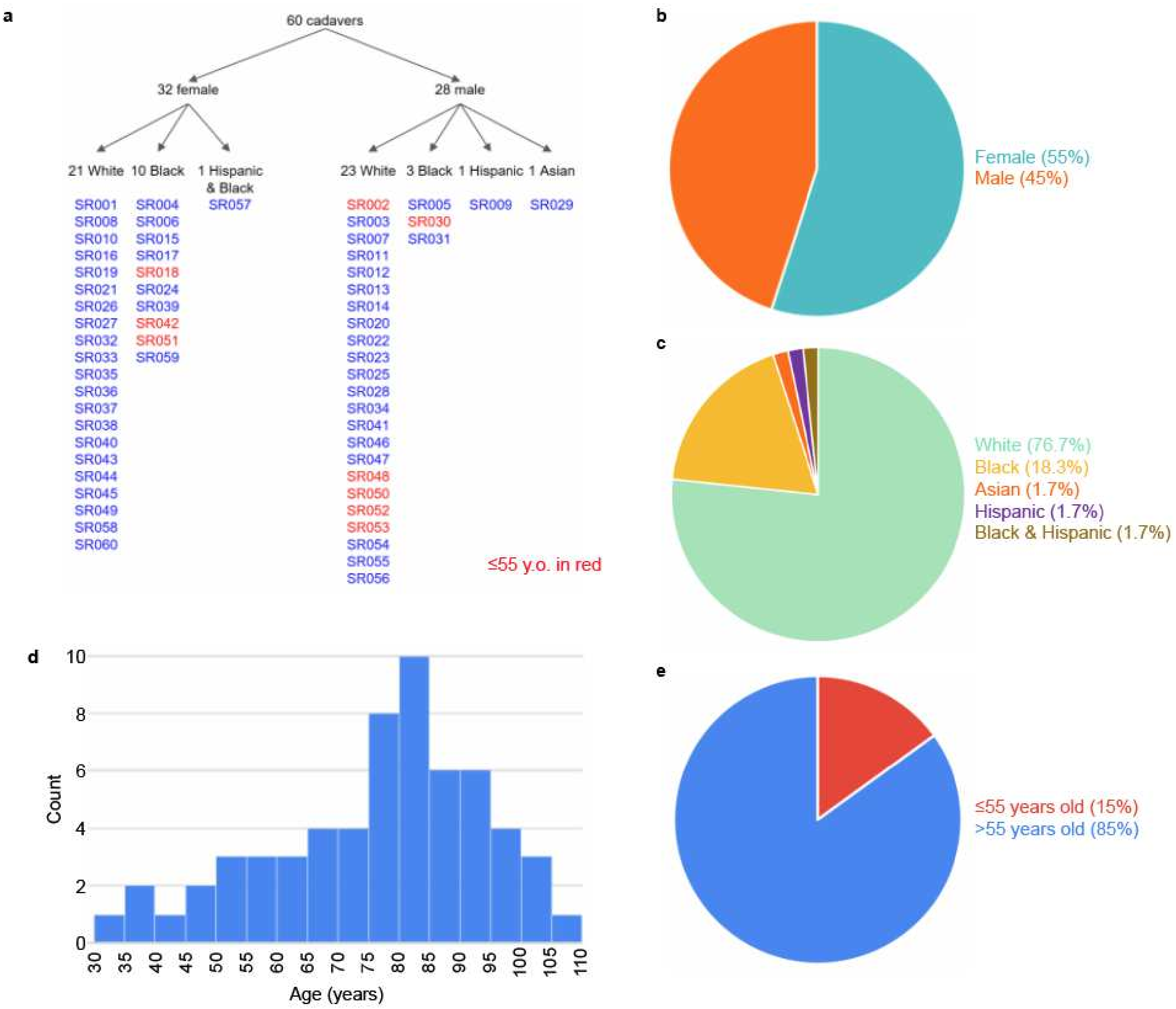

Figure 6. Cadaver demographics.

Supplement 2

Term list

Table 6. List of standardized anatomical terms, including anatomical directions, vagal structures, non-vagal nerves and branches, anatomical levels on the vagus nerve, and anatomical landmarks (bony and soft tissue). *The terms are approximately ordered from superior to inferior.*

Term	Modifier (if applicable)
**Anatomical directions**	
superior	none
inferior	none
medial	none
lateral	none
anterior	none
posterior	none
proximal	none
distal	none
**Vagal structures**	
cranial nerve rootlets	right/left
vagus nerve	right/left
branch to unknown target	right/left
branch to unknown nerve tissue	right/left
broken branch	right/left
jugular ganglion	right/left
nodose ganglion	right/left
meningeal branch	right/left
auricular branch	right/left
branch of auricular branch to tympanic membrane	right/left
branch of auricular branch to facial nerve	right/left
branch of auricular branch to floor of external acoustic meatus and pinna	right/left
branch to glossopharyngeal nerve	right/left
branch to superior root of ansa cervicalis	right/left
branch to inferior root of ansa cervicalis	right/left
branch to ansa cervicalis	right/left
branch to omohyoid muscle	right/left
branch to sternohyoid muscle	right/left
branch to spinal accessory nerve	right/left
branch to hypoglossal nerve	right/left
branch to C1 spinal nerve	right/left
branch to C2 spinal nerve	right/left
branch to C3 spinal nerve	right/left
branch to C4 spinal nerve	right/left
branch to C5 spinal nerve	right/left
branch to trachea	right/left
pharyngeal branch	right/left
branch of pharyngeal branch	right/left
branch to pharyngeal branch	right/left
branch to pharyngeal nerve plexus	right/left
branch to branch to pharyngeal nerve plexus	right/left
branch of pharyngeal branch to carotid sinus nerve	right/left
fusion with cervical sympathetic trunk	right/left
fusion with superior cervical ganglion	right/left
branch to cervical sympathetic trunk	right/left

branch to branch to cervical sympathetic trunk	right/left
branch to thoracic sympathetic trunk	right/left
branch to branch to thoracic sympathetic trunk	right/left
branch to superior cervical ganglion	right/left
branch to cervical cardiac nerve	right/left
branch to common carotid artery	right/left
branch to internal jugular vein	right/left
branch to great vessels	right/left
branch to internal carotid artery	right/left
branch to external carotid artery	right/left
branch to carotid bifurcation	right/left
branch to carotid sinus nerve	right/left
branch of superior laryngeal nerve	right/left
branch of superior laryngeal nerve to cervical sympathetic trunk	right/left
branch of superior laryngeal nerve to carotid bifurcation	right/left
branch of superior laryngeal nerve to carotid sinus nerve	right/left
superior laryngeal nerve	right/left
internal branch of superior laryngeal nerve	right/left
branch of internal branch of superior laryngeal nerve	right/left
external branch of superior laryngeal nerve	right/left
branch of external branch of superior laryngeal nerve	right/left
branch to superior laryngeal nerve	right/left
branch to internal branch of superior laryngeal nerve	right/left
branch to external branch of superior laryngeal nerve	right/left
branch to branch to superior laryngeal nerve	right/left
superior branch of internal branch of superior laryngeal nerve	right/left
middle branch of internal branch of superior laryngeal nerve	right/left
inferior branch of internal branch of superior laryngeal nerve	right/left
branch to cricothyroid muscle	right/left
branch to sternothyroid muscle	right/left
branch to branch to cricothyroid muscle	right/left
cervical vagus nerve	right/left
cervical vagus to vagus branch	right/left
branch to cervical vagus to vagus branch	right/left
cervical vagus to thoracic vagus branch	right/left
cervical branch to lymph node	right/left
thoracic branch to lymph node	right/left
cervical thoracic vagus nerve	right/left
thoracic vagus nerve	right/left
thoracic vagus to vagus branch	right/left
branch to thoracic vagus to vagus branch	right/left
branch to thymus	right/left
cervical cardiopulmonary branch	right/left
cervical cardiac branch	right/left
branch to cervical cardiac branch	right/left
thoracic cardiopulmonary branch	right/left
thoracic cardiac branch	right/left
branch to thoracic cardiac branch	right/left
recurrent laryngeal nerve	right/left
branch to recurrent laryngeal nerve	right/left
branch to branch to recurrent laryngeal nerve	right/left
cardiac branch of recurrent laryngeal nerve	right/left
branch of recurrent laryngeal nerve	right/left
branch to branch of recurrent laryngeal nerve	right/left
pulmonary branch	right/left
branch to esophagus	right/left

tracheoesophageal branch	right/left
branch to esophageal plexus	right/left
sub branch	right/left
esophageal plexus trunk	anterior/posterior
abdominal esophageal branch	anterior/posterior
branch to diaphragm	right/left
esophageal plexus cardiopulmonary branches	none
esophageal plexus cardiopulmonary branch	none
esophageal plexus cardiac branches	none
esophageal plexus cardiac branch	none
esophageal plexus pulmonary branches	none
esophageal plexus pulmonary branch	none
esophageal plexus branches	none
esophageal plexus branch	none
esophageal plexus tracheoesophageal branch	none
esophageal plexus diaphragmatic branches	none
esophageal plexus diaphragmatic branch	none
esophageal plexus gastric branch	none
esophageal plexus hepatic branch	none
esophageal plexus	anterior/posterior
periarterial plexus	none
abdominal trunk	anterior/posterior
esophageal plexus communicating branch	none
anterior esophageal plexus communicating branch	none
posterior esophageal plexus communicating branch	none
branch of esophageal plexus communicating branch	none
branch of anterior esophageal plexus communicating branch	none
branch of posterior esophageal plexus communicating branch	none
gastric branches	none
gastric branches of anterior trunk	none
gastric branches of posterior trunk	none
gastric branch	none
gastric branch of anterior trunk	none
gastric branch of posterior trunk	none
pyloric branches	none
pyloric branches of anterior trunk	none
pyloric branches of posterior trunk	none
pyloric branch	none
pyloric branch of anterior trunk	none
pyloric branch posterior trunk	none
abdominal branches	anterior/posterior
abdominal branch	anterior/posterior
hepatic branches	none
hepatic branches of anterior trunk	none
hepatic branches of posterior trunk	none
hepatic branch	none
hepatic branch of anterior trunk	none
hepatic branch of posterior trunk	none
pancreatic branches	none
pancreatic branches of anterior trunk	none
pancreatic branches of posterior trunk	none
pancreatic branch	none
pancreatic branch of anterior trunk	none
pancreatic branch of posterior trunk	none
celiac branch	none
celiac branch of anterior trunk	none

celiac branch of posterior trunk	none
small intestinal branches	none
small intestinal branches of anterior trunk	none
small intestinal branches of posterior trunk	none
small intestinal branch	none
small intestinal branch of anterior trunk	none
small intestinal branch of posterior trunk	none
large intestinal branch	none
large intestinal branch of anterior trunk	none
large intestinal branch of posterior trunk	none
diaphragmatic branches	none
diaphragmatic branches of anterior trunk	none
diaphragmatic branches of posterior trunk	none
diaphragmatic branch	none
diaphragmatic branch of anterior trunk	none
diaphragmatic branch of posterior trunk	none
cystic branch	none
cystic branch of anterior trunk	none
cystic branch of posterior trunk	none
subesophageal plexus	none
**Non-vagal nerves and branches**	
glossopharyngeal nerve	right/left
branch of glossopharyngeal nerve	right/left
branch of glossopharyngeal nerve to carotid sinus nerve	right/left
pharyngeal branch of glossopharyngeal nerve	right/left
spinal accessory nerve	right/left
branch of spinal accessory nerve	right/left
hypoglossal nerve	right/left
branch of hypoglossal nerve	right/left
branch of hypoglossal nerve to superior root of ansa cervicalis	right/left
cervical sympathetic trunk	right/left
thoracic sympathetic trunk	right/left
branch of cervical sympathetic trunk	right/left
branch of thoracic sympathetic trunk	right/left
superior cervical ganglion	right/left
middle cervical ganglion	right/left
stellate ganglion	right/left
carotid sinus nerve	right/left
branch of carotid sinus nerve	right/left
ansa cervicalis	right/left
branch of ansa cervicalis	right/left
branch of ansa cervicalis to omohyoid muscle	right/left
branch of ansa cervicalis to infrahyoid muscles	right/left
branch of ansa cervicalis to sternohyoid muscle	right/left
branch of ansa cervicalis to sternothyroid muscle	right/left
superior root of ansa cervicalis	right/left
branch of superior root of ansa cervicalis	right/left
branch of superior root of ansa cervicalis to sternohyoid muscle	right/left
branch of superior root of ansa cervicalis to sternothyroid muscle	right/left
branch of superior root of ansa cervicalis to carotid bifurcation	right/left
branch of superior root of ansa cervicalis to omohyoid muscle	right/left
inferior root of ansa cervicalis	right/left
branch of inferior root of ansa cervicalis	right/left
branch of inferior root of ansa cervicalis to omohyoid muscle	right/left
great auricular nerve	right/left

branch of great auricular nerve	right/left
lesser occipital nerve	right/left
transverse cervical nerve	right/left
branch of transverse cervical nerve	right/left
supraclavicular nerve	right/left
branch of supraclavicular nerve	right/left
facial nerve	right/left
marginal mandibular branch of facial nerve	right/left
branch of marginal mandibular branch of facial nerve	right/left
buccal branch of facial nerve	right/left
cervical branch of facial nerve	right/left
branch of cervical branch of facial nerve	right/left
cervical cutaneous nerve	right/left
cervical spinal nerve	right/left
C1 spinal nerve	right/left
C2 spinal nerve	right/left
C3 spinal nerve	right/left
C4 spinal nerve	right/left
C5 spinal nerve	right/left
C6 spinal nerve	right/left
C7 spinal nerve	right/left
C8 spinal nerve	right/left
branch of C1 spinal nerve	right/left
branch of C2 spinal nerve	right/left
branch of C3 spinal nerve	right/left
branch of superior cervical ganglion to the carotid sinus nerve	right/left
aortic depressor nerve	right/left
pharyngeal plexus	right/left
superior cardiac nerve	right/left
ansa subclavia	right/left
unknown nerve tissue	right/left

**Levels on the vagus nerve**	
level of superior border of jugular foramen	right/left
level of inferior border of jugular foramen	right/left
level of C1 transverse process	right/left
level of greater horn of hyoid	right/left
level of laryngeal prominence	right/left
level of angle of the mandible	right/left
level of carotid bifurcation	right/left
level of superior border of the clavicle	right/left
level of jugular notch	right/left
level of sternal angle	right/left
level of tracheal bifurcation	right/left
level of 1 cm superior to start of esophageal plexus	right/left
level of esophageal hiatus	anterior/posterior
level of aortic hiatus	anterior/posterior
level of aortic hiatus on pancreatic branch	
level of aortic hiatus on pyloric branch	
level of aortic hiatus on gastric branch	anterior/posterior

**Anatomical landmarks (bony)**	
mental protuberance	none
mastoid process	right/left
jugular notch of the manubrium	none
pubic symphysis	none

acromion process	right/left
sternal angle	none
greater horn of the hyoid bone	right/left
lateral edge of the body of the hyoid bone	right/left
angle of the mandible	right/left
transverse process of C1 vertebra	right/left
C7 spinous process	none
iliac crest	right/left
center of iliac crest	none
jugale	right/left
infraorbital foramen	right/left
external occipital protuberance	none
center of zygomatic arch	right/left
zygomatic process	right/left
**Anatomical landmarks (soft tissue)**	
tip of nose	none
laryngeal prominence	none
esophageal hiatus	none
tracheal bifurcation	none
apex of the heart	none
ligamentum arteriosum	none
aortic hiatus	none
carotid bifuration	right/left
carotid arteries	right/left
cardiac notch of the stomach	
**Other structures**	
blood vessel	
medulla	right/left
common carotid artery	right/left
internal carotid artery	right/left
external carotid artery	right/left
esophagus	
body of the stomach	
pylorus	
lesser curvature	
greater curvature	
pyloric sphincter	
lymph node	
fascia	

Supplement 3

Anatomical levels

Table 7. Anatomical levels on the vagus nerve that are painted. *See the landmarks defined in* ***Table 8****.*

**Name of landmark**	**Modifier of landmark (if applicable)**	**Section(s) of the vagus nerve that contain the level**	**Description**
Superior border of the jugular foramen	Right, left	Cervical right, cervical left	The location on the vagus nerve or cranial nerve rootlets that emerges superiorly from the jugular foramen.
Inferior border of the jugular foramen	Right, left	Cervical right, cervical left	The location on the vagus nerve that emerges inferiorly from the jugular foramen.
Transverse process of C1 vertebra	Right, left	Cervical right, cervical left	The estimated axial level of the C1 vertebra transverse process landmark on the ipsilateral side vagus nerve.
Angle of the mandible	Right, left	Cervical right, cervical left	The estimated axial level of the angle of the mandible landmark on the ipsilateral side vagus nerve.
Greater horn of the hyoid bone	Right, left	Cervical right, cervical left	The estimated axial level of the tip of the greater horn of the hyoid landmark on the ipsilateral side vagus nerve.
Carotid bifurcation	Right, left	Cervical right, cervical left	The estimated axial level of the carotid bifurcation landmark on the ipsilateral side vagus nerve.
Laryngeal prominence	N/A	Cervical right, cervical left	The estimated axial level of the laryngeal prominence landmark on both the right and left vagus nerves.
Superior border of the clavicle	Right, left	Cervical right, cervical left	The location on the vagus nerve that is in the same axial plane as the ipsilateral clavicle.
Jugular notch of the manubrium	N/A	Thoracic right, thoracic left	The estimated axial level of the jugular notch of the manubrium landmark on both the right and left vagus nerves.
Sternal angle	N/A	Thoracic right, thoracic left	The estimated axial level of the sternal angle landmark on both the right and left vagus nerves.
Tracheal bifurcation	N/A	Thoracic right, thoracic left	The estimated axial level of the tracheal bifurcation landmark on both the right and left vagus nerves.
1 cm superior to the start of esophageal plexus	N/A	Thoracic right, thoracic left	The estimated location that is 1 cm superior to the start of the esophageal plexus (as determined by the dissector) on the right and left vagus nerves.
Esophageal hiatus on the anterior trunk	N/A	Esophageal plexus	The estimated axial level of the esophageal hiatus landmark on the anterior trunk of the vagus.
Esophageal hiatus on posterior trunk	N/A	Esophageal plexus	The estimated axial level of the esophageal hiatus landmark on the posterior trunk of the vagus.

Esophageal hiatus (not used for all cadavers)	N/A		The estimated axial level of the esophageal hiatus landmark on the vagus nerve in the esophageal plexus that is not a clear anterior or posterior trunk of the vagus.
Aortic hiatus	N/A	Subesophageal plexus	The estimated axial level of the aortic hiatus landmark on the closest vagus nerve structure to the landmark or midline. There may be more than one location if there are multiple vagal branches close to the midline.
Inferior border of the cranium (not used for all cadavers)	Right, left	Cervical right, cervical left	The estimated axial level on the vagus nerve where it emerges inferiorly from the cranium.

Table 8. List of gross anatomical landmarks identified to estimate the locations of levels for painting on the vagus nerve. *All skeletal landmarks are directly on the bone, and all soft tissue superficial to the bone has been removed, unless noted with an asterisk (*) where the landmark could not be visually identified and was instead palpated.*

**Name**	**Modifier (if applicable)**	**Description**
Superior border of the jugular foramen	Right, left	Identify the jugular foramen, which is the hole in the cranium through which the vagus nerve, glossopharyngeal nerve, spinal accessory nerve, internal jugular vein, and inferior petrosal sinus pass to exit the skull. Locate the superior-most edge.
Inferior border of the jugular foramen	Right, left	Identify the jugular foramen (see row above) and locate the inferior-most edge.
Transverse process of C1 *	Right, left	The most lateral edge of the transverse process of the first cervical vertebra. There may be some tissue superficial to this location.
Angle of the mandible	Right, left	The most lateral surface of the vertex of the angle of the mandible (formed by the junction of the body and ramus of the mandible). The mandibles are removed during dissection and are manually held in place when needed.
Greater horn of the hyoid bone *	Right, left	The most superior edge of the tubercles of the greater horns of the hyoid bone from the lateral side. There may be some tissue (e.g., muscle) superficial to this location.
Carotid bifurcation	Right, left	The most inferior and lateral point on the “Y” shape formed by the bifurcation of the common carotid artery on the vasculature. The carotid bifurcation is moved significantly during dissection but pinned in its approximate pre-dissection location when needed.
Laryngeal prominence		The most anterior location that is formed by the laryngeal prominence of the thyroid cartilage. There may be some tissue (e.g., muscle) superficial to this location.
Superior border of the clavicle	Right, left	The most superior border of the clavicular bone.
Jugular notch of the manubrium		The most superior edge in the notch formed in the midline of the manubrium. The chest wall is removed during dissection and is laid in place when needed.
Sternal angle		The most anterior surface in the sagittal midline of the junction between the manubrium and the body of the sternum. The chest wall is removed during dissection and is laid in place when needed.
Tracheal bifurcation		The most anterior surface of the superior edge of the inside of the upside-down “Y” shape formed by the tracheal bifurcation. The trachea is moved significantly during dissection but manually held in its approximate pre-dissection location when needed.
Esophageal hiatus		The most anterior margin of the esophageal hiatus. Pin(s) is/are placed in the lateral part of the esophagus to mark the site of the hiatus when the diaphragm is incised during dissection.
Aortic hiatus		The most anterior margin of the aortic hiatus. Pin(s) is/are placed in the aorta to mark the approximate site of the hiatus when the diaphragm is incised during dissection.

Supplement 4

Anatomical landmarks

Table 9. List of skeletal anatomical landmarks identified when 3D nerve tracing.

Name	Modifier (if applicable)	Description
Mental protuberance	N/A	The most anterior vertex in the sagittal midline of the mental protuberance on the bone.
Jugale	Right, left	The most lateral point in the inner notch is formed by the “L” shape of the jugale on the bone.
Infraorbital foramen	Right, left	The skin is cut to reveal the infraorbital foramen with the infraorbital nerve. The nerve is partially scraped and removed. The tip of the stylus is placed inside the infraorbital foramen approximately 1-2 mm deep.
Acromion process	Right, left	The most anterior edge of the vertex is formed by the acromion process on the bone.
Lateral edge of the body of the hyoid bone	Right, left	Palpate to feel the hyoid bone, and locate the anterior side of the center of the lateral border or edge of the hyoid bone on the skin.
Greater horn of the hyoid bone	Right, left	Palpate to locate the most superior edge of the tubercles of the greater horns of the hyoid on the skin, from an anterolateral angle.
Angle of the mandible	Right, left	The vertex is formed by the angle of the mandible (postero-inferiorly), on the most lateral surface of the bone. The mandibles are removed during dissection and are reattached temporarily for tracing.
Transverse process of C1	Right, left	Palpate to locate the most lateral edge of the transverse process on the skin.
Jugular notch of the manubrium	N/A	The most superior edge in the notch formed in the midline of the manubrium on the bone. The chest wall is removed during tracing and is reattached temporarily for tracing.
Sternal angle	N/A	The junction between the manubrium and the body of the sternum, on the most anterior surface in the sagittal midline of the sternum. The chest wall is removed during tracing and is reattached temporarily for tracing.
Pubic symphysis	N/A	The most anterior surface of the center of the pubic symphysis.
External occipital protuberance	N/A	The most posterior vertex formed by the projection on the occipital bone. It can only be identified with the cadaver in the prone position.
Zygomatic process	Right, left	The most lateral point on the zygomatic process.
Center of the zygomatic arch	Right, left	The approximate center of the zygomatic arch, on the superior-lateral surface of the bone.
Mastoid process	Right, left	The most inferior vertex formed by the mastoid process.
C7 spinous process	N/A	The most posterior vertex of the C7 spinous process. It can only be palpated with the cadaver in the prone position.
Iliac crest	Right, left	The most superior point of the iliac crest. It can only be palpated with the cadaver in the prone position.
Center of iliac crest	N/A	The approximate location in the sagittal midline of the cadaver that is between the two most superior points on the right and left iliac crests. It can only be located with the cadaver in the prone position.

Table 10. List of soft tissue anatomical landmarks used for 3D nerve tracing.

Name	Modifier (if applicable)	Description
Tip of nose	N/A	The tip of the vertex of the nose in the sagittal midline of the body, on the skin.
Laryngeal prominence	N/A	The most anterior location on the tip of the vertex that is formed by the laryngeal prominence of the thyroid cartilage. Located by palpating on the skin.
Carotid bifurcation	Right, left	The most inferior and lateral point on the saddle shape formed by the carotid arteries (e.g., the inside of the “Y” shape) on the vasculature.
Tracheal bifurcation	N/A	The most anterior surface of the inferior edge of the notch is formed by the tracheal bifurcation. The trachea is moved significantly during dissection but held in its approximate pre-dissection location during tracing.
Apex of the heart	N/A	The vertex formed by the apex of the heart that points in the left-anterior direction in most individuals. The heart is moved significantly during dissection but held in its approximate pre-dissection location during tracing.
Ligamentum arteriosum	N/A	The most anterolateral surface in the approximate center of the ligament that attaches the aorta to the pulmonary artery. The heart is moved significantly during dissection but held in its approximate pre-dissection location during tracing.
Esophageal hiatus	N/A	The most anterior margin of the esophageal hiatus. This is always an estimated location as it is difficult to identify, the diaphragm collapses post-mortem, and structures have been removed during dissection.
Aortic hiatus	N/A	The most anterior margin of the aortic hiatus. This is always an estimated location as it is difficult to identify, the diaphragm collapses post-mortem, and structures have been removed during dissection.

Supplement 5

REVA (CWRU/Duke) Consortium

**Leadership**	
Andrew J. Shoffstall	Department of Biomedical Engineering, Case Western Reserve University, Cleveland, OH, USA, 44106; APT Center, Louis Stokes Cleveland Department of Veterans Affairs Medical Center, Cleveland, OH, USA, 44106
Nicole A. Pelot	Department of Biomedical Engineering, Duke University, Durham, NC, USA, 27708
**Anatomy**	
Andrew R. Crofton (team PI)	Department of Anatomy, Case Western Reserve University, Cleveland, OH, USA, 44106; Department of Pathology and Cell Biology, University of South Florida, Tampa, FL, USA, 33612
Brandon A.S. Brunsman	Department of Anatomy, Case Western Reserve University, Cleveland, OH, USA, 44106
Leina Lunasco	Department of Anatomy, Case Western Reserve University, Cleveland, OH, USA, 44106
Noa B. Nuzov	Department of Biomedical Engineering, Case Western Reserve University, Cleveland, OH, USA, 44106
Tatiana Pascol	Department of Anatomy, Case Western Reserve University, Cleveland, OH, USA, 44106
Rebecca Prince	Department of Anatomy, Case Western Reserve University, Cleveland, OH, USA, 44106
Ashley Onabiyi	Department of Anatomy, Case Western Reserve University, Cleveland, OH, USA, 44106
Zeyna Samba	Department of Anatomy, Case Western Reserve University, Cleveland, OH, USA, 44106
Valerie H. Lam	Department of Biomedical Engineering, Case Western Reserve University, Cleveland, OH, USA, 44106; Department of Anatomy, Case Western Reserve University, Cleveland, OH, USA, 44106
Katharine Workman	Department of Anatomy, Case Western Reserve University, Cleveland, OH, USA, 44106
Sophie K. Scherer	Department of Anatomy, Case Western Reserve University, Cleveland, OH, USA, 44106
Morgan L. Griffith	Department of Anatomy, Case Western Reserve University, Cleveland, OH, USA, 44106
Steven O'Neill	Department of Anatomy, Case Western Reserve University, Cleveland, OH, USA, 44106
Marissa L. Brigger	Department of Anatomy, Case Western Reserve University, Cleveland, OH, USA, 44106
Matthew E. Davis	Department of Biomedical Engineering, Case Western Reserve University, Cleveland, OH, USA, 44106
Ann A. Yonas	Department of Biomedical Engineering, Case Western Reserve University, Cleveland, OH, USA, 44106
Logan Adams	Department of Anatomy, Case Western Reserve University, Cleveland, OH, USA, 44106
Shriya Minocha	Department of Biomedical Engineering, Duke University, Durham, NC, USA, 27708
Sydney Rubin	Department of Anatomy, Case Western Reserve University, Cleveland, OH, USA, 44106
Sara Bokhari	Department of Anatomy, Case Western Reserve University, Cleveland, OH, USA, 44106
Nicholas J. Ogrinc	Department of Anatomy, Case Western Reserve University, Cleveland, OH, USA, 44106
Grace Misiunas	Department of Anatomy, Case Western Reserve University, Cleveland, OH, USA, 44106
Anya Malhotra	Department of Anatomy, Case Western Reserve University, Cleveland, OH, USA, 44106
Anjali K. Bhuthpur	Department of Anatomy, Case Western Reserve University, Cleveland, OH, USA, 44106
Sarah H. Ouda	Department of Biomedical Engineering, Duke University, Durham, NC, USA, 27708
Nikki Pillai	Department of Anatomy, Case Western Reserve University, Cleveland, OH, USA, 44106
Jessi Villafuerte	Department of Anatomy, Case Western Reserve University, Cleveland, OH, USA, 44106
Megan Settell	Department of Neurological Surgery, University of Wisconsin-Madison, Madison, WI
Anabella Santos	Department of Biomedical Engineering, Duke University, Durham, NC, USA, 27708
Cooper Lahti	Department of Anatomy, Case Western Reserve University, Cleveland, OH, USA, 44106
Kathryn Turk	Department of Biomedical Engineering, Duke University, Durham, NC, USA, 27708
Jasper Reimers	Department of Anatomy, Case Western Reserve University, Cleveland, OH, USA, 44106; Department of Biomedical Engineering, Case Western Reserve University, Cleveland, OH, USA, 44106
**MRI**	
Ari Blitz (team PI)	Department of Radiology, Case Western Reserve University and University Hospitals Cleveland Medical Center, Cleveland, OH, USA, 44106
Daniel A. Herzka	Department of Radiology, Case Western Reserve University and University Hospitals Cleveland Medical Center, Cleveland, OH, USA, 44106
Chris Flask	Department of Radiology, Case Western Reserve University and University Hospitals Cleveland Medical Center, Cleveland, OH, USA, 44106
Andrew R. Crofton	Department of Anatomy, Case Western Reserve University, Cleveland, OH, USA, 44106; Department of Pathology and Cell Biology, University of South Florida, Tampa, FL, USA, 33612
Shruti Kumari	Department of Radiology, Case Western Reserve University and University Hospitals, Cleveland Medical Center, Cleveland, OH, USA, 44106
Noa B. Nuzov	Department of Biomedical Engineering, Case Western Reserve University, Cleveland, OH, USA, 44106
Mridhula Muthukumar	Department of Radiology Case Western Reserve University and University Hospitals Cleveland Medical Center, Cleveland, OH, USA, 44106
Goksel Sali	Department of Radiology Case Western Reserve University and University Hospitals Cleveland Medical Center, Cleveland, OH, USA, 44106
Michael Markley	University Hospitals Cleveland Medical Center, Cleveland, OH, USA 44106
Owen R. Emch	Department of Radiology Case Western Reserve University and University Hospitals Cleveland Medical Center, Cleveland, OH, USA, 44106
Ella N. Smullen	Department of Radiology Case Western Reserve University and University Hospitals Cleveland Medical Center, Cleveland, OH, USA, 44106
Max Sheng	Department of Radiology Case Western Reserve University and University Hospitals Cleveland Medical Center, Cleveland, OH, USA, 44106
**CT**	
Noa B. Nuzov	Department of Biomedical Engineering, Case Western Reserve University, Cleveland, OH, USA, 44106
**MicroCT**	
Andrew J. Shoffstall (team PI)	Department of Biomedical Engineering, Case Western Reserve University, Cleveland, OH, USA, 44106; APT Center, Louis Stokes Cleveland Department of Veterans Affairs Medical Center, Cleveland, OH, USA, 44106

Jichu Zhang	Department of Biomedical Engineering, Case Western Reserve University, Cleveland, OH, USA, 44106
Aniruddha Upadhye	Department of Biomedical Engineering, Case Western Reserve University, Cleveland, OH, USA, 44106
Constantinos Tsipsis	Department of Biomedical Engineering, Case Western Reserve University, Cleveland, OH, USA, 44106
Megan C. Thompson	Department of Biomedical Engineering, Case Western Reserve University, Cleveland, OH, USA, 44106
Anandakumar Shunmugavel	Department of Biomedical Engineering, Case Western Reserve University, Cleveland, OH, USA, 44106
Talya A. Jeter	Department of Biomedical Engineering, Case Western Reserve University, Cleveland, OH, USA, 44106
Noa B. Nuzov	Department of Biomedical Engineering, Case Western Reserve University, Cleveland, OH, USA, 44106
Havisha Kalpatthi	North Allegheny Senior High School, Wexford, PA, USA, 15090; N/A
Maryse Lapierre-Landry	Department of Biomedical Engineering, Case Western Reserve University, Cleveland, OH, USA, 44106
Justin Chin	Department of Biomedical Engineering, Case Western Reserve University, Cleveland, OH, USA, 44106
Renil Contractor	Department of Anatomy, Case Western Reserve University, Cleveland, OH, USA, 44106
Jay Warrier	Department of Biomedical Engineering, Duke University, Durham, NC, USA, 27708; N/A
**Histology**	
Andrew J. Shoffstall (team PI)	Department of Biomedical Engineering, Case Western Reserve University, Cleveland, OH, USA, 44106; APT Center, Louis Stokes Cleveland Department of Veterans Affairs Medical Center, Cleveland, OH, USA, 44106
Jennifer J. Coleman	Department of Biomedical Engineering, Case Western Reserve University, Cleveland, OH, USA, 44106
Aniya Hartzler	Department of Biomedical Engineering, Case Western Reserve University, Cleveland, OH, USA, 44106
Eleana Cintron	Department of Biomedical Engineering, Case Western Reserve University, Cleveland, OH, USA, 44106
Talya A. Jeter	Department of Biomedical Engineering, Case Western Reserve University, Cleveland, OH, USA, 44106
Mallika Singh	Department of Biomedical Engineering, Case Western Reserve University, Cleveland, OH, USA, 44106
Jennifer D'Silva	Department of Biomedical Engineering, Case Western Reserve University, Cleveland, OH, USA, 44106
Proapa Islam	Department of Biomedical Engineering, Case Western Reserve University, Cleveland, OH, USA, 44106
Jichu Zhang	Department of Biomedical Engineering, Case Western Reserve University, Cleveland, OH, USA, 44106
Miarasa Steele (DiNuoscio)	Department of Biomedical Engineering, Case Western Reserve University, Cleveland, OH, USA, 44106; University Hospitals, Anatomic Pathology & Histology, Cleveland Ohio 44106
Rico Washington	Department of Biomedical Engineering, Case Western Reserve University, Cleveland, OH, USA, 44106
Akyla Collins	Department of Biomedical Engineering, Case Western Reserve University, Cleveland, OH, USA, 44106; Cleveland Clinic, Pathology, Cleveland Ohio, 44195
Youjoung Kim	Department of Biomedical Engineering, Case Western Reserve University, Cleveland, OH, USA, 44106
Logan Chu	Department of Computer Science, Trinity College of Arts and Sciences, Duke University, Durham, North Carolina, United States, 27708
Harrison G. Fazzone	Department of Computer Science, Trinity College of Arts and Sciences, Duke University, Durham, North Carolina, United States, 27708
Evan Merzon	Department of Computer Science, Trinity College of Arts and Sciences, Duke University, Durham, North Carolina, United States, 27708
Crystal Soong	Department of Computer Science, Trinity College of Arts and Sciences, Duke University, Durham, North Carolina, United States, 27708
Qianyi Chen	Department of Computer and Data Sciences, Case School of Engineering, Case Western Reserve University, Cleveland, OH, USA, 44106
Shayan Pandit	Department of Biomedical Engineering, Duke University, Durham, NC, USA, 27708
Heather Quinones	Department of Biomedical Engineering, Case Western Reserve University, Cleveland, OH, USA, 44106; Cleveland Metro Schools
Olivia Rentsch	Department of Biomedical Engineering, Case Western Reserve University, Cleveland, OH, USA, 44106
Alyssa Schellhouse	Department of Biomedical Engineering, Case Western Reserve University, Cleveland, OH, USA, 44106
Viveka Rabara	Colleage of Arts and Sciences, Case Western Reserve University, Cleveland, OH, USA, 44106
Yuvha Karthikeyan	Department of Biomedical Engineering, Case Western Reserve University, Cleveland, OH, USA, 44106; Solon High School, Solon, OH, USA, 44139
Karim Elsharkawy	Department of Biomedical Engineering, Case Western Reserve University, Cleveland, OH, USA, 44106
Keith Brennan	Department of Biomedical Engineering, Case Western Reserve University, Cleveland, OH, USA, 44106
**3D-MUSE**	
Michael W. Jenkins (team PI)	Department of Biomedical Engineering, Case Western Reserve University, Cleveland, OH, USA, 44106; FES Center, Louis Stokes Cleveland VA Medical Center, Cleveland, OH, USA; Department of Pediatrics, Case Western Reserve University, Cleveland, OH, 44106
David L. Wilson (team PI)	Department of Biomedical Engineering, Case Western Reserve University, Cleveland, OH, USA, 44106; Department of Radiology, Case Western Reserve University, Cleveland, OH 44106
James Seckler	Department of Biomedical Engineering, Case Western Reserve University, Cleveland, OH, USA, 44106
Naomi Joseph	Department of Biomedical Engineering, Case Western Reserve University, Cleveland, OH, USA, 44106
Jocelyn Chin	Department of Biomedical Engineering, Case Western Reserve University, Cleveland, OH, USA, 44106
Proapa Islam	Department of Biomedical Engineering, Case Western Reserve University, Cleveland, OH, USA, 44106
Ian Marshall	Department of Biomedical Engineering, Case Western Reserve University, Cleveland, OH, USA, 44106
Quan D. Tran	Department of Biomedical Engineering, Case Western Reserve University, Cleveland, OH, USA, 44106
Juri Moon	Department of Biomedical Engineering, Case Western Reserve University, Cleveland, OH, USA, 44106
Suyash Moore	Department of Biomedical Engineering, Case Western Reserve University, Cleveland, OH, USA, 44106
Kaichen Yu	Department of Biomedical Engineering, Case Western Reserve University, Cleveland, OH, USA, 44106
Brooke M. Husar	Department of Biomedical Engineering, Case Western Reserve University, Cleveland, OH, USA, 44106
Nathan Petranka	Department of Biomedical Engineering, Case Western Reserve University, Cleveland, OH, USA, 44106
Gillian Leigh Strout	Department of Biomedical Engineering, Case Western Reserve University, Cleveland, OH, USA, 44106
Ananya Veerubhotla	Department of Biomedical Engineering, Case Western Reserve University, Cleveland, OH, USA, 44106
Vo Linh Chi Dao	Department of Biomedical Engineering, Case Western Reserve University, Cleveland, OH, USA, 44106
Jiwoo Kim	Department of Biomedical Engineering, Case Western Reserve University, Cleveland, OH, USA, 44106
Bathlomew A. Ebika	Department of Biomedical Engineering, Case Western Reserve University, Cleveland, OH, USA, 44106
Tanishka Isaac	Department of Biomedical Engineering, Case Western Reserve University, Cleveland, OH, USA, 44106

Grace R. Garbonick	Department of Biomedical Engineering, Case Western Reserve University, Cleveland, OH, USA, 44106
Urmika Ghosh	Department of Biomedical Engineering, Case Western Reserve University, Cleveland, OH, USA, 44106
Chaitanya Kolluru	Department of Biomedical Engineering, Case Western Reserve University, Cleveland, OH, USA, 44106
**Modeling**	
Nicole A. Pelot (team PI)	Department of Biomedical Engineering, Duke University, Durham, NC, USA, 27708
Ian Baumgart	Department of Biomedical Engineering, Duke University, Durham, NC, USA, 27708
Warren M. Grill	Department of Biomedical Engineering, Duke University, Durham, NC, USA, 27708; Department of Electrical and Computer Engineering, Duke University, Durham, NC, USA, 27708; Department of Neurobiology, Duke University, Durham, NC, USA, 27708; Department of Neurosurgery, Duke University, Durham, NC, USA, 27708
Princess Tara Zamani	Department of Biomedical Engineering, Duke University, Durham, NC, USA, 27708
**Data**	
Mark F. Beno (team PI)	Cleveland Institute for Computational Biology, Case Western Reserve University, Cleveland, OH, USA, 44106
Audrey Lynn (team PI)	Cleveland Institute for Computational Biology, Case Western Reserve University, Cleveland, OH, USA, 44106
Sunah Song	Cleveland Institute for Computational Biology, Case Western Reserve University, Cleveland, OH, USA, 44106
Beverly Koepf	Cleveland Institute for Computational Biology, Case Western Reserve University, Cleveland, OH, USA, 44106
Robert Lanese	Cleveland Institute for Computational Biology, Case Western Reserve University, Cleveland, OH, USA, 44106
**Scientific Advisory Board**	
Warren M. Grill	Department of Biomedical Engineering, Duke University, Durham, NC, USA, 27708; Department of Electrical and Computer Engineering, Duke University, Durham, NC, USA, 27708; Department of Neurobiology, Duke University, Durham, NC, USA, 27708; Department of Neurosurgery, Duke University, Durham, NC, USA, 27708
Kip Ludwig	Department of Neurological Surgery, University of Wisconsin-Madison, Madison, WI, USA, 53792; Department of Surgery, University of Wisconsin-Madison, Madison, WI, USA, 53792; Wisconsin Institute of Translational Neuroengineering (WITNe), University of Wisconsin-Madison, Madison, WI, USA, 53792
Gene Civillico	
Megan Settell	Department of Neurological Surgery, University of Wisconsin-Madison, Madison, WI
Jonathan Z. Baskin	APT Center, Louis Stokes Cleveland Department of Veterans Affairs Medical Center, Cleveland, OH; Department of Biomedical Engineering, Case Western Reserve University, Cleveland, OH, USA, 44106; Department of Surgery, Louis Stokes Cleveland VA Medical Center, Cleveland, OH, USA; School of Medicine, Case Western Reserve University, Cleveland, OH, USA
Kevin Otto	Weldon School of Biomedical Engineering, Purdue University, West Lafayette, IN, USA, 47907
**Additional Contributors**	
David Nethery	Department of Biomedical Engineering, Case Western Reserve University, Cleveland, OH, USA, 44106
Julie C. Savage	Department of Biomedical Engineering, Case Western Reserve University, Cleveland, OH, USA, 44106
Kenneth J. Gustafson	Department of Biomedical Engineering, Case Western Reserve University, Cleveland, OH, USA, 44106; FES Center, Louis Stokes Cleveland VA Medical Center, Cleveland, OH, USA
Jeff Capadona	APT Center, Louis Stokes Cleveland VA Medical Center, Cleveland, OH, USA; Department of Biomedical Engineering, Case Western Reserve University, Cleveland, OH, USA, 44106
